# A qualitative study of experiences of institutional objection to medical assistance in dying in Canada: ongoing challenges and catalysts for change

**DOI:** 10.1186/s12910-023-00950-9

**Published:** 2023-09-21

**Authors:** Eliana Close, Ruthie Jeanneret, Jocelyn Downie, Lindy Willmott, Ben P White

**Affiliations:** 1 Australian Centre for Health Law Research, Faculty of Business and Law, Queensland University of Technology, GPO Box 2434, Brisbane, QLD 4000 Australia; 2 Health Law Institute, Faculties of Law and Medicine, Dalhousie University, Halifax, Canada

**Keywords:** Assisted dying, Medical assistance in dying, Euthanasia, Assisted suicide, Institutional objection, Conscientious objection, Patient experience, Health professional experience

## Abstract

**Background:**

In June 2016, Canada legalized medical assistance in dying (MAiD). From the outset, some healthcare institutions (including faith-based and non-faith-based hospitals, hospices, and residential aged care facilities) have refused to allow aspects of MAiD onsite, resulting in patient transfers for MAiD assessments and provision. There have been media reports highlighting the negative consequences of these “institutional objections”, however, very little research has examined their nature and impact.

**Methods:**

This study reports on findings from 48 semi-structured qualitative interviews conducted with MAiD assessors and providers, MAiD team members (working to coordinate care and lead MAiD programs in institutions and health authorities), and family caregivers on their experiences with institutional objection. Participants were recruited from the Canadian provinces of British Columbia, Ontario, and Nova Scotia. Data were analyzed using inductive thematic analysis.

**Results:**

Themes identified were: (1) basis for institutional objection (with objections commonly rooted in religious values and a particular philosophy of palliative care); (2) scope of objection (demonstrating a wide range of practices objected to); (3) lack of transparency regarding institutional position; (4) impacts on patients; (5) impacts on health practitioners; and (6) catalysts for change. Participants reported that many institutions’ objections had softened over time, lessening barriers to MAiD access and adverse impacts on patients and health practitioners. Participants attributed this positive change to a range of catalysts including advocacy by health practitioners and family members, policymaking by local health authorities, education, and relationship building. Nevertheless, some institutions, particularly faith-based ones, retained strong objections to MAiD, resulting in forced transfers and negative emotional and psychological impacts on patients, family members, and health practitioners.

**Conclusions:**

This paper adds to the limited evidence base about the impacts of institutional objection and can inform practical and regulatory solutions in Canada and abroad. Reform is needed to minimize the negative impacts on patients, their caregivers, and health practitioners involved in MAiD practice.

**Supplementary Information:**

The online version contains supplementary material available at 10.1186/s12910-023-00950-9.

## Background

A growing number of jurisdictions have legalized medical assistance in dying (MAiD) (elsewhere known as euthanasia and physician-assisted suicide, or voluntary assisted dying) [1, 2]. MAiD is now potentially available to many millions of people worldwide [1]. Despite this trend, barriers exist which restrict the ability of individuals to access MAiD. One such barrier is “institutional conscientious objection” or “institutional objection” [3, 4] by hospitals, hospices, and residential aged care facilities, which seek to exclude aspects of MAiD on conscientious or religious grounds.

In Canada, where MAiD has been legal since 2016, [5]^1^ approximately 3% of all deaths annually are through MAiD (3.3% in 2021) [6]. To be eligible for MAiD, two independent medical or nurse practitioners must assess the patient and determine that they meet the relevant legislative criteria. Both physician-administered and oral MAiD are permitted by law, but to date, the vast majority of MAiD deaths have been administered by a physician or nurse practitioner, rather than administered by patients themselves [6].^2^ More than half of all MAiD provisions have occurred in a healthcare institution such as a hospital (28.6% of MAiD deaths in 2021), palliative care facility (19.6%), or residential aged care facility (6.1%) [6]. Some institutions facilitate or passively permit MAiD, while others refuse to be involved or prohibit it happening onsite [7–9].

Due to the constitutional division of powers in Canada, institutional objection is dealt with on a provincial/territorial level [10].^3^ As a result, the legal position of objecting institutions varies across Canadian provinces and territories [7]. In Quebec, all “institutions” (defined to include hospitals and residential and long-term care facilities, but not palliative care hospices) must offer end-of-life care, including MAiD [11]. In Nova Scotia, all facilities operated by the Nova Scotia Health Authority (which owns and operates all hospitals) must provide or allow access to MAiD [12]. Likewise, in Prince Edward Island, the provincial health department indicates eligible individuals can receive MAiD education, assessments, and procedures at the location of their choice, including “any publicly funded health care institution in the community, health care centre or hospital.” [13] In the remaining provinces, some degree of institutional objection is protected either by legislation or through agreements between the provincial government and faith-based healthcare institutions [7]. For example, in British Columbia, institutions which have over 50% of their beds publicly funded are required by government policy to allow MAiD assessment and provision with an important exception [14] – faith-based institutions can prohibit MAiD in their facilities under a broad “Master Agreement” between the province and the Denominational Health Care Facilities Association [15].

Like the Canadian regulatory position, the ethical literature on institutional objection lacks consensus. While the ability of individual health practitioners to refuse to participate in MAiD is a well-recognised albeit not universally accepted ethical principle (which is also reflected in laws and policies), [16] institutional objection is more contested. Some argue that institutions cannot claim to have a conscience since they are “bricks and mortar” and cannot suffer moral injury like individual health practitioners can [4, 17]. Others argue that institutions with a distinct ethos, such as a religious organization, can claim a shared set of values akin to an individual’s conscience, which should be protected [4, 18]. Still others argue that institutional objection is justifiable not on the basis of conscience, but rather as a matter of self-governance [19]. Yet, given the considerable harms to patients that can result from institutional objection, detractors suggest it should be prohibited, or at least curtailed [3, 4, 20, 21]. Institutional objections, they say, can restrict the availability of certain health services for many people and “will almost always wipe out access for huge numbers of people because institutions serve huge numbers of people” [22].

There is very limited data on the prevalence of institutional objection to MAiD in Canada. In a retrospective chart review of MAiD deaths in the Vancouver Coastal Health Authority in British Columbia from 17 June 2016–17 June 2019, Wiebe et al. found 9.5% (42 cases) involved a forced transfer [8]. Alberta Health Services reported that of 842 persons who accessed MAiD in a major hospital facility between 17 June 2016 and 30 September 2020, 15% (124) were transferred for the procedure. Of those transferred, 87% (109) were transferred from a faith-based facility (13% of persons who accessed MAiD in Alberta) (this data is no longer available on the Alberta Health Services website) [23]. Providence Health Care, a non-profit Catholic health care organization that operates hospitals and residential aged care facilities in British Columbia, has indicated that from June 2016 (when MAiD was legalized) to June 2023, 402 patients made formal MAiD requests and 131 patients were transferred elsewhere for MAiD provision [24].

A small but growing body of research in Canada and internationally has demonstrated a range of harms to patients caused by institutional objections [3, 8, 9, 25, 26]. Institutions have refused to provide patients with information about MAiD, and have refused to permit assessments and provisions onsite, resulting in some patients being transferred out of a facility [3, 8, 9, 25, 26]. These transfers have caused patients to experience additional pain, and psychological, emotional, and psychosocial suffering [3, 8, 24–26, 28]. In some circumstances, institutional objections have blocked patients’ access altogether, such as when there was no other entity to receive a transfer of the patient or the transfer was physically unbearable for the patients or otherwise impossible [8, 28]. Some studies have also found more insidious effects of institutional objection, which can adversely affect a patient’s end-of-life experience. In a study of the perceptions of health providers, patients and family members from a Saskatchewan regional health authority, Brown et al. found that participants perceived institutional policies prohibiting MAiD as creating barriers to access and challenges in navigating institutional procedures [25]. In particular, participants reported being unclear who in an institution was “safe to approach when accessing, receiving, and providing care.” [25] Some family caregivers in the Australian state of Victoria also reported institutional objection to MAiD adversely affecting trust in clinical advice [26].

There is also some emerging evidence internationally that institutional objections also cause harms to health professionals, and reduce willingness to participate in MAiD [9, 27–29]. Physicians have described structural and emotional challenges from faith-based institutions refusing to allow entry to undertake MAiD assessments and provisions onsite, practising privileges not being honoured, significant travel needed as assessments cannot be carried out onsite, uncertainty caused by lack of protocols and policies, and onerous reporting requirements [9, 28, 29]. Nurses in Belgium have reported that a lack of professional support constrained their ability to represent the patient’s interests [30]. Health professionals who do not share the institution’s position experience moral distress when compelled to act against their values as a result of an institutional position [8, 25, 30]. Volunteer witnesses also described concerns about forced transfers and challenges when witnessing MAiD requests in faith-based institutions [31].

There are limited studies addressing institutional objections to MAiD in Canada to date [8, 9, 25, 31]. Existing evidence has largely arisen as a minor subset of findings from wider studies reporting on experiences with the MAiD system more broadly, with the exception of Wiebe et al’s 2021 examination of forced and chosen transfers before and during the COVID-19 pandemic [8]. Additionally, existing evidence on institutional objection has mostly been from studies conducted in the first year or two of MAiD being legal in Canada, during a period when the MAiD system was still developing. This article examines experiences of institutional objection in Canada, six years after MAiD was legalized with the passage of Bill C-14 in June 2016. Its purpose is to report on perceptions of the impacts of institutional objection to MAiD on patients and health practitioners in contemporary practice now that the system in Canada has become more established. It aims to identify how institutional objections are experienced and the factors that have shaped practice over time.

## Methods

### Study design

This study is part of a broader comparative international project investigating factors that shape decision-making about MAiD in Canada, Australia and Belgium, to inform an optimal holistic model of regulation [32]. A discrete area of investigation was perceptions of the impact of institutional objection to MAiD in practice. This paper reports on the Canadian experience from data collected through semi-structured interviews with family caregivers of persons who sought MAiD, and physicians, nurse practitioners, and other health professionals who are involved in MAiD as assessors and providers or as members of institutional or health authority MAiD teams. The method is reported in accordance with the consolidated criteria for reporting qualitative studies (COREQ) [33].

### Sampling and recruitment

For feasibility, since the study aimed to capture the specific regulatory context relevant to MAiD, which varies by province/territory, [34] the research team selected three target provinces to recruit from: British Columbia, Ontario, and Nova Scotia. These provinces were selected to provide diversity in geography, size, and population distribution. Individuals were eligible to participate in the study if they were over 18 and involved in decision-making about MAiD in the target provinces in one (or more) of three roles:

1. *Individuals and family caregivers*. Persons seeking MAiD who had initiated the process were eligible to participate, whether or not they had been assessed for MAiD or found eligible. Since patients suffering from grievous and irremediable conditions can be a difficult cohort to recruit, [35] particularly because they are by definition experiencing enduring and intolerable suffering, we also sought perspectives of family caregivers of patients who had experienced the MAiD process. Family caregivers were eligible to participate if they had supported a family member through the MAiD process (whether or not they had been found eligible) and could therefore speak to the patient’s experiences. These participants were initially recruited using social media (Twitter and the project website) and through emails from Dying with Dignity Canada (the leading national patient advocacy, education, and support group for MAiD).
2. *MAiD assessors/providers*. Physicians and nurse practitioners were eligible to participate if they had acted as a MAiD assessor (assessing patient eligibility) or as a provider of MAiD (assessing the patient’s eligibility and administering or prescribing the medication). These participants were initially recruited using social media (Twitter and the project website), and through the Canadian Association of MAiD Assessors and Providers (CAMAP) (the national professional organization for health professionals involved in MAiD).
3. *MAiD team members.* The third group was comprised of individuals who had a professional role as a member of a MAiD program (typically involved in activities including program management, care coordination, education, and research) either with a health authority or within an institution. These participants were initially recruited using social media (Twitter and the project website), and through CAMAP.

Since this investigation is part of a broader study on optimal regulation of MAiD, as noted in the study design, advertisements were framed broadly, seeking participants with experience of decision-making about MAiD and views on the impact of regulation (including law, policy, and procedures). Initially, all participants were recruited using convenience sampling, based on who had responded to preliminary recruitment efforts, as detailed above. We subsequently used purposive sampling to enhance diversity in terms of sex, location (metropolitan/regional), and patient, provider, and MAiD team experiences. Snowball sampling was also used to identify additional participants, also targeting diversity in experiences and location. Recruitment ceased when the research team determined there was sufficient “information power” to meet the study aims [36].

### Data collection

The research team developed semi-structured interview guides for each interview cohort (Additional files 1, 2, 3, 4). Interviews covered a range of issues as part of the broader study on decision-making about MAiD mentioned above, and institutional objection was raised using prompts if participants did not raise it themselves. The key open-ended prompts for each interview cohort were:

- *Interviews with patients or family caregivers (regarding patients in an institution)*: “Did the facility facilitate access to MAiD or was it a barrier to access?”;
- *Interviews with MAiD assessors/providers*: “Have you experienced any issues with institutions which object to MAiD at any stage of the MAiD process?”; and.
- *Interviews with MAiD team members*: “Have you been involved with addressing institutional objections and/or transfers?”

When a participant discussed institutional objection (either explicitly using that term or others, e.g. “forced transfers”), follow up questions explored issues including: the nature of the objection (e.g. where and at what stage of the process it arose, and how it was communicated); what the impact of the objection was (e.g. how it affected the patient, family members, and staff); and any action taken to respond to the objection. The interviewers used techniques including paraphrasing and summarizing in interviews to check understanding of the participants’ views and experiences.

Patient and family caregiver interviews were conducted by RJ (with JD present for 2 interviews and EC for 10 interviews to pilot the interview guide and provide feedback as part of RJ’s PhD research training). Interviews with MAiD assessors/providers and MAiD team members were conducted by EC (with JD present for 3 initial interviews to refine the interview guide). One interview was conducted jointly by EC and RJ as the participant was both a family caregiver and MAiD assessor/provider.

Interviews were conducted between 6 October 2021 and 9 August 2022 using Zoom videoconferencing software. All participants provided informed consent prior to the interview. For all family caregiver interviews, the patient whose experience they were sharing had died, and so patient consent was not sought. EC and RJ (and where applicable, JD) debriefed after interviews, and maintained reflexive journals. Interview audio was recorded using Zoom and professionally transcribed verbatim. Participants were given the opportunity to add to, clarify or amend their transcript.

### Analysis

All transcripts were uploaded to NVivo (release 1.6.1, QSR International) for analysis. EC and RJ identified the transcripts that described experiences with institutional objection. Institutional objection was defined as occurring when a participant perceived an institution (including a hospital, hospice, aged-care facility, or long-term care facility) objected to some or all aspects of MAiD on the basis of values (rather than purely logistical considerations), including when this objection was not explicitly stated. For example, this included participants that reported access to MAiD was affected because of an institution’s religious affiliation or due to interactions with staff, even if the institution did not have an explicit position against MAiD.

Once these transcripts were identified, EC and RJ selected 10 transcripts (5 involving family caregivers, 4 with MAiD assessors/providers and 1 with a MAiD team member) and each independently inductively coded all extracts addressing institutional objection, using Braun and Clarke’s reflexive thematic analysis [36]. After this initial coding, EC and RJ discussed coding approaches to achieve a richer understanding of the data, [37] and refined the codes. EC and RJ then each independently coded the remainder of the transcripts. To enrich analysis, EC and RJ identified areas of convergence and divergence, particularly between data collected from each of the three distinct participant groups and provinces. Using this coding and considering the reflexive journals, EC and RJ developed themes and sub-themes, which were discussed and iteratively refined by all authors after reviewing the data.

## Results

### Sample description

Seventy interviews were conducted for the broader project on MAiD decision-making: 31 with family caregivers; one with a patient; 32 with MAiD assessors/providers (25 physicians and 7 nurse practitioners); and 11 with members of MAiD teams at health authorities and institutions. Five participants had overlapping roles: four MAiD team members were also MAiD assessors/providers, and one MAiD assessor/provider was also a family caregiver. Two interviews with family caregivers involved two participants, at the interviewees’ request (e.g. a child of a deceased parent and their spouse).

In 48 of the 70 interviews, participants discussed institutional objection: 40 interviews described direct experiences, while 8 interviews solely involved participants’ perceptions of institutional objection more generally. The proportion of participants discussing institutional objection by participant role is set out in Table 1.

**Table 1:** Interviews in which participants discussed institutional objection

Role	Number of interviews: Total sample	Number of interviews: Institutional objection sample (% of total sample)	Number of interviews with direct experiences of institutional objection (% of institutional objection sample)	Examples of direct experiences of institutional objection
Family caregivers	31	12 (39%)	9 (75%)	• Supporting a patient who experienced an institutional objection (6 interviews) • Experience in participant’s capacity as a healthcare worker (2 interviews) • Experience as a volunteer witness for MAiD (1 interview)
Patient	1	1 (100%)	-	• N/A^4^
MAiD assessors/providers	32	31 (97%)	27 (87%)	• Negotiating access to MAiD with objecting institutions • Conducting MAiD assessments in objecting institutions • Caring for patients who experienced forced transfers
MAiD team members	11	9 (82%)	9 (100%)	• Managing care coordination with objecting institutions • Education • Policy development and implementation
Total^5^	70	48 (69%)	40 (83%)	-

This subset of 48 interviews discussing institutional objection, which was analyzed for this study, ranged from 50 to 203 min (median of 94 min). Table 2 sets out participant demographics. Table 3 sets out characteristics of the 6 patients who family caregivers described experienced an institutional objection (characteristics of patients reported on by family caregivers from the broader study are also included to facilitate comparison).

**Table 2:** Characteristics of participants (total sample and institutional objection sample)

Characteristics	Total sample:Number (%)	Institutional objection sample:Number (%)	Institutional objection sample (direct experiences):Number (%)
*Family caregivers (n = 33 in total sample, 14 in institutional objection sample, 11 with direct experiences)* ^6^			
Gender			
Female	25 (76%)	11 (79%)	2 (18%)
Male	8 (24%)	3 (21%)	9 (82%)
Age			
Median (interquartile range)	60 (51–72)	61.5 (55.5–70)	60 (50.5–62.5)
Province			
British Columbia	9 (27%)	3 (21%)	2 (18%)
Ontario	17 (52%)	9 (64%)	8 (73%)
Nova Scotia	6 (18%)	2 (14%)	1 (9%)
Other^7^	1 (3%)	-	-
Relationship to patient			
Child/child-in-law	16 (48%)	8 (57%)	8 (73%)
Spouse/partner	12 (36%)	5 (36%)	2 (18%)
Parent	2 (6%)	1 (7%)	1 (9%)
Close friend	2 (6%)	-	-
Niece	1 (3%)	-	-
Relationship to patient			
Child/child-in-law	16 (48%)	8 (57%)	8 (73%)
Spouse/partner	12 (36%)	5 (36%)	2 (18%)
Parent	2 (6%)	1 (7%)	1 (9%)
Close friend	2 (6%)	-	-
Niece	1 (3%)	-	-
***Patient (n = 1 in total sample, 1 in institutional objection sample)*** ^8^			
Gender			
Female	-	-	-
Male	1 (100%)	1 (100%)	-
Province			
British Columbia	1 (100%)	1 (100%)	-
Ontario	-	-	-
Nova Scotia	-	-	-
Illness, disease or disability for which MAiD is sought			
Cancer	-	-	-
Neurological condition	-	-	-
Cardiovascular condition	-	-	-
Respiratory condition	-	-	-
Other condition	1 (100%)	1 (100%)	-
***MAiD assessors/providers (n = 32 in total sample, 31 in institutional objection sample, 27 with direct experiences)***			
Gender			
Female	21 (66%)	20 (65%)	18 (67%)
Male	11 (34%)	11 (35%)	9 (33%)
Age			
Median (interquartile range)	50.5 (42–61)	50 (42–61)	52 (42–62)
Province			
British Columbia	10 (31%)	10 (32%)	8 (30%)
Ontario	15 (47%)	14 (45%)	13 (48%)
Nova Scotia	7 (22%)	7 (23%)	6 (22%)
Population Centre and Rural Area Classification [51]			
Large urban population centre (> 100,000)	17 (53%)	16 (52%)	14 (52%)
Medium population centre (30,000–99,999)	5 (16%)	5 (16%)	3 (11%)
Small population centre (1,000–29,999)	6 (19%)	6 (19%)	6 (22%)
Rural area	4 (13%)	4 (13%)	4 (15%)
Type of assessor/provider			
Physician	25 (78%)	24 (77%)	21 (78%)
Nurse practitioner	7 (22%)	7 (23%)	6 (22%)
Main clinical specialty			
Family medicine	14 (44%)	14 (45%)	13 (48%)
Primary care	6 (19%)	5 (16%)	5 (19%)
Palliative care	4 (13%)	4 (13%)	2 (7%)
Psychiatry	3 (9%)	3 (10%)	2 (7%)
Anaesthesia	1 (3%)	1 (3%)	1 (4%)
Geriatric medicine	1 (3%)	1 (3%)	1 (4%)
Internal medicine	1 (3%)	1 (3%)	1 (4%)
Neurology	1 (3%)	1 (3%)	1 (4%)
Oncology	1 (3%)	1 (3%)	1 (4%)
Practice setting			
Community only	16 (50%)	16 (52%)	14 (52%)
Hospital only	5 (16%)	5 (16%)	5 (19%)
Multiple settings (including community, hospital, hospice)	11 (34%)	10 (32%)	8 (30%)
Years of experience in health care			
Median (interquartile range)	20.5 (11-33.8)	21 (11–35)	21 (11–37)
Number of MAiD cases as assessor and/or provider			
Median (interquartile range)	112.5 (51.3-337.5)	125 (50–350)	200 (38.75–387.5)
***MAiD team members (n = 11 in total sample, 9 in institutional objection sample, 9 with direct experiences)***			
Gender			
Female	7 (64%)	6 (67%)	6 (67%)
Male	4 (36%)	3 (33%)	3 (33%)
Age			
Median (interquartile range)	49 (41–54)	49 (42–58)	49 (42–58)
Province			
British Columbia	4 (36%)	4 (44%)	4 (44%)
Ontario	4 (36%)	2 (22%)	2 (22%)
Nova Scotia	3 (27%)	1 (33%)	1 (33%)
Population Centre and Rural Area Classification [51]			
Large urban population centre (> 100,000)	8 (73%)	6 (67%)	6 (67%)
Medium population centre (30,000–99,999)	2 (18%)	2 (22%)	2 (22%)
Small population centre (1,000–29,999)	-	-	-
Rural area	1 (9%)	1 (11%)	1 (11%)
Setting			
Health authority	8 (73%)	8 (89%)	8 (89%)
Institution	3 (27%)	1 (11%)	1 (11%)

^6^ Note: 31 interviews were conducted with 33 family caregivers (2 interviews with family caregivers each involved 2 participants; these are included in both the total sample and institutional objection sample)

^7^ One family caregiver was based internationally but spoke about patient experiences in British Columbia and another province

^8^ Since only a single patient was recruited, patient age is not reported to protect patient privacy

**Table 3:** Characteristics of patients discussed by family caregivers (total sample and patients who experienced an institutional objection)

Characteristic	Total sample (n = 32)^9^ Number (%)	Patients who experienced aninstitutional objection (n = 6) Number (%)
Gender		
Female	18 (56%)	4 (67%)
Male	14 (44%)	2 (33%)
Age		
Median (interquartile range)	74.5 (66-81.25)	69.5 (66.3–72.8)
Province		
British Columbia	10 (31%)	2 (33%)
Ontario	14 (44%)	2 (33%)
Nova Scotia	6 (19%)	1 (17%)
Other^10^	2 (6%)	1 (17%)
Population Centre and Rural Area Classification [51]		
Large urban population centre (> 100,000)	18 (56%)	3 (50%)
Medium population centre (30,000–99,999)	6 (19%)	1 (17%)
Small population centre (1,000–29,999)	4 (13%)	-
Rural area	4 (13%)	2 (33%)
Place of death		
Residence	17 (53%)	2 (33%)
Hospital	8 (25%)	-
Hospice	1 (3%)	1 (17%)
Long-term care facility	4 (13%)	2 (33%)
Assisted living facility	1 (3%)	-
Other	1 (3%)	1 (17%)
Patient status at time of interview		
*MAiD death*	29 (91%)	6 (100%)
*Non-MAiD death*		
Assessed as eligible for MAiD but lost capacity	2 (6%)	-
Assessed as ineligible for MAiD	1 (3%)	-
Illness, disease or disability for which MAiD was sought		
Cancer	20 (63%)	3 (50%)
Neurological condition	7 (22%)	2 (33%)
Cardiovascular condition	2 (6%)	-
Respiratory condition	2 (6%)	-
Other condition	1 (6%)	1 (17%)
Year of death		
2016	1 (3%)	-
2017	5 (16%)	3 (50%)
2018	10 (31%)	1 (17%)
2019	3 (9%)	1 (17%)
2020	5 (16%)	-
2021	8 (25%)	1 (17%)

^9^ One interview from the total sample of 31 family caregiver interviews discussed 2 patient experiences

^10^ A participant based in one of the three target provinces described a patient experience in another province

Participants reported institutional objections from a range of faith-based and non-faith-based institutions including hospitals, palliative care units, hospices, and long-term care facilities. While institutional objection occurred in all provinces, MAiD assessors/providers and MAiD team members from Ontario commented on more ongoing challenges with institutional objection than those in British Columbia and Nova Scotia.

We identified six overarching themes: (1) basis of institutional objection; (2) scope of objection; (3) transparency of position; (4) impacts on patients; (5) impacts on health practitioners; and (6) catalysts for institutional change. Additional illustrative quotes for each theme are provided in Table 4.

**Table 4:** Additional illustrative quotes regarding institutional objection by theme

Theme	Illustrative quotes (additional examples to what is provided in the text)
1. Basis for institutional objection	*Religious values* “So all the facilities over the country who claim to not participate – mostly Catholic, some other private ones – do it on the basis of their faith…” (MAiD assessor/provider 24) “…they were looking at it from the perspective of we are a Jewish hospital and Jewish laws don’t believe in medically assisted death.” (Family caregiver 11) *Philosophy of palliative care* “… our hospice isn’t even a religious organization … and they wanted none of it, and the palliative physicians didn’t want any part of it.” (MAiD assessor/provider 3) “…it’s a huge barrier. We can’t go into hospice. In fact, hospice tells patients when they’re interviewing them that if they are considering MAID they will not be allowed to come into hospice.” (MAiD assessor/provider 23) *Influence of key stakeholder* “I think it’s completely at the board level. Like all things, governance decides everything institutionally. And I think what’s keeping the governance from accepting it is probably … perceptions around reputation risk.” (MAiD team member 9) “…[the director] is the one that puts a kibosh on [i.e. puts a stop to] anything remotely concerning MAID … The only inpatient palliative care unit we have … we still have forced transfers, we still have – people aren’t even allowed to be assessed on site. They have to move for both assessments and provision.” (MAiD assessor/provider 1) “…they’re all funded with public money [faith-based institutions] and the patients and the staff don’t reflect those same values.” (MAiD assessor/provider 24)
2. Scope of refusal	*Blanket refusal* “So, the [hospital] today, I went to the parking lot to do the assessment. They won’t let me in the building.” (MAiD assessor/provider 26) *Shift to allow assessments* “I think that’s what they came down to, it’s the act of actually ending a life which is what their religion says you can’t do. Talking about it, finding someone eligible, until you’ve actually ended life you haven’t done something which is against God’s law.” MAiD assessor/provider 6) “They said they would allow the assessment but not the provision, and then they upheld that. …we did think, especially as a provincially-funded health facility, that they would be required to abide by provincial health laws. But, in fact, religion trumped the law.” (Family caregiver 28) *Other aspects of the MAiD process* Information: “I still think there is an active access issue because providers in that institution are not telling people that it’s an option.” (MAiD assessor/provider 11) “…who feels empowered to even ask about MAID in a Catholic institution in which they know it’s not allowed? And whose conversation about MAID gets passed the nurse who says “No, we don’t do that here,” or the resident who says “No, we don’t do that here,”? Like the persistence required to even get an assessment or have a conversation with your MRP [Most Responsible Physician] about MAID in a Catholic institution must be enormous, and it is enormous.” (MAiD assessor/provider 15) IV access: “Some of our nursing agencies won’t even put an IV in for that procedure…” (MAiD assessor/provider 18) Witnessing: “… we’ve had witnessings where the patients have to leave the hospital and get the forms signed on the sidewalk because they won’t do it inside the hospital.” (Family caregiver 8) (also a volunteer witness for MAiD) “I have a patient in [rural area] … He had no one to witness his form. He receives homecare on a daily basis, metastatic [cancer]. … a nurse visiting him daily … a palliative care coordinator … a palliative nurse practitioner who’s visiting from [city]. All of them are forbidden by their agencies to witness. … I messaged these people, because they’re all friendly people of mine, and I said ‘Please take this to your supervisor. This is now legal. You are a paid caregiver. You are allowed to sign this legally.’ ‘Oh, no, I’m sorry, it is our policy.’ The [hospital], they have a policy, nobody who’s an employee of the [hospital] is allowed to sign this form. I said ‘You’ve created a barrier now. You’ve added a barrier to the law’…” (MAiD assessor/provider 26) *Discretionary decisions* “They … came up with a thousand and one excuses not to follow the rules and to – they seemed bound and determined to avoid it at all costs. … They were obstructive right until the bitter end.” (Family caregiver 24a) “… after a year of allowing forms and assessments, they [the hospice] wouldn’t allow him to sign his form on the premises and said he would have to leave the premises. Which is a huge area. It’s a hospital and nursing home and a hospice in one area. So I said to them ‘Are you telling me you want this guy to go in his electric scooter, in the rain and winter, and go a kilometre and a half to leave the property? Which newspaper would you like to talk to?’” (MAiD assessor/provider 24) “I had another patient transferred to hospice and I got someone on the phone, and they said, ‘We’ll let you in the building.’ ‘Okay, thanks.’ But I’m not sure if that’s a policy thing. They said, ‘We will not provide MAID.’ It’s on their application to the hospice, the patients have to sign that they will not even discuss MAID when they are admitted to a hospice.” (MAiD assessor/provider 26) “We’ve done very well at working with our institutional conscientious objections. … in some places sometimes they have to be transferred back, but other places, believe it or not, actually will allow us to assess and provide.” (MAiD team member 9) *Locations with little to no problems* “I’m very lucky where we are. The only encounter that I did have was early on when one of our facilities did not have a policy about MAID in place at all one way or the other.” (MAiD assessor/provider 12) “… I know it happens in other provinces. I know other [specialists] just say “Well, I work in a Catholic place, so I don’t have to do it, like I have never been involved.“ I must say I’ve been to the hospice a few times in Halifax and one of the first times I was there, there was obviously a nurse that was uncomfortable with me being there and I got a bit of a cold shoulder. But that happened once and never again.” (MAiD assessor/provider 2)
3. Lack of transparency regarding institutional position	*Lack of transparency regarding institutional position* “They pretended – or they said this was the first time they’d ever faced this situation in the home. So they came up with a thousand and one excuses not to follow the rules and to – they seemed bound and determined to avoid it at all costs.” (Family caregiver 24b) “She’d been at [institution] for coming on three years. So, basically, as soon as we found out that it was a law now, that we could have that, we started working on that process … So she’d been approved for a little while and then I think she decided that we would give it a couple of months, and in that couple of months we tied up everything … And then we found out that [institution] would not allow her to have the procedure, her provision in her home.” (Family caregiver 28) “They were unorganised, both in terms of the technical procedures and what you had to go through, but also and more important culturally equipped to deal with it. Their staff didn’t know how to react. They had no protocol of what to tell or not to tell other residents on the floor who knew that something was going on.” (Family caregiver 12) “On the face of it, it doesn’t seem like that big of an issue. Like, okay, no problem, you don’t allow assessments or assisted deaths, they’ll just go to another spot. But like patients don’t know. They don’t know that these healthcare institutions have those regulations because, at least in [province], they’re not being upfront about it. So a patient just walks into a hospital thinking that it’s a hospital. So then they’re there and now they have to leave, but if they leave then you’re withdrawing them from their care team that they already know and trust … you’re causing pain and suffering on the transfer. … They’re applying for MAID to end their enduring suffering and you’re adding to their enduring suffering by having them leave for the assessment and the death.” (MAiD team member 6)
4. Impacts on patients	*Physical pain and other suffering caused by forced transfers* “… they’re still being transferred to a different part of the hospital. … there’s still that stigma that you still have to go to a different part of the hospital. … So it’s not perfect. It’s pretty good, but it’s not perfect.” (MAiD assessor/provider 10) “…the transfer was very uncomfortable. So, unfortunately that was really bad. To have someone move off of a location where they’ve lived for 10 years in a long-term care facility, that is just ridiculous and hard.” (MAiD assessor/provider 12) “I had a case where he was in the hospice and he had to be transported out. … to be transported to this place, the poor man had been in so much pain.” (MAiD assessor/provider 17) *Constrained choice* “But where it comes into play for me … is when I’m seeing patients from a palliative care perspective and I ask them what their goals are and they say ‘I want to stay home as long as possible, but if things get too bad I want to have MAID and if things get too bad I want to go to an inpatient setting.’ Well, you can’t have both. You have to make a choice. So if things get too bad and you need to be admitted because your family’s not coping or your symptoms aren’t being managed well enough, then you basically give up MAID. You don’t have to. But the reality is if you’re sick enough to need inpatient care, it’s not going to be to your best interest to then be transferred to [major hospital] to have the procedure done at that point. So again I’ve never dissuaded somebody from making the choice that’s right for them, but they need to be informed and the reaction I get is always, ‘Are you kidding me?’ So it’s disheartening.” (MAiD assessor/provider 1) “[The patient had been] … languishing for like three weeks after having made a MAID request because they happened to find themselves, by virtue of an ambulance, choosing [a Catholic hospital] over a secular institution. They just happened to find themselves in a place that didn’t affirm their autonomy to make decisions around their end-of-life care. So yeah, it’s unconscionable, it’s bonkers, it’s unjust, it’s nonsensical. It causes distress for patients, it causes distress for clinicians.” (MAiD assessor/provider 15) *Compromised access* “… I might be able to provide for them at this hospital, I’d get emergency privileges to do it, but they wouldn’t have the people around them who had been really caring for them in a very tender and supportive way for a long time. So, I’ve had patients who have then made the decision to just let nature take its course and they missed out on MAID because of that.” (MAiD assessor/provider 5) “The transfer services aren’t - I mean they’re not made for MAID. We don’t book - we don’t have a system where you arrive at 10:00 and then you pick up the patient. They come when they’re ready. So then that means that families are disconnected from each other in the last hours of the person’s life because the family’s left to go to the new spot but the patient is still waiting over here. We’ve had instances where the patient’s lost capacity because they got too much pain medication on route…” (MAiD team member 6) *Mitigating factors for individual patients* “We have had some patients … where the physician has gone in unbeknownst to the staff, done a MAID assessment and then the patient’s been transferred home to have the procedure.” (MAiD assessor/provider 1) “She got MAID is what happened, because I like barrelled right through that. Because … the woman asked for MAID. I am her physician. It is my job to make sure she gets it.” (MAiD assessor/provider 14) “I did do an assessment there. I didn’t ask permission. I just went in as a visitor.” (MAiD assessor/provider 23) “We were running a secret MAiD operation … Behind their backs.” (Family caregiver 12) “And the home came up with basically, the same form with their letterhead on top, and said my mum has to fill all of this out. And I put my foot down and said ‘No, absolutely not. This government form is effectively the same thing as your form. You’re welcome to have a photocopy of the government form, otherwise leave us alone.’” (Family caregiver 24a)
5. Impacts on health professionals	*Emotional impacts* “I had one patient, they [the staff] went to the media and they went to the police to try to have me arrested.” (MAiD assessor/provider 3) “…the people on the frontline, the doctors and nurses on the frontline are loving and wonderful people who suffer great moral distress when they have to do this [participate in forced transfers].” (MAiD assessor/provider 17) “I said, you know, that I would continue to do assessments without any problem, and they threatened me with a College report. And I said ‘Oh, I’ve had lots of talks with the Registrar of the College … we’ve had good, long conversations about this and they do not support your position at all. They believe that doctors should be able to see patients and talk and they don’t support hospital privileging interfering with this.’ So then we made an agreement.” (MAiD assessor/provider 22) “We [publicly advocated] saying ‘… we are very concerned that there is a lack of knowledge and access for this.’ So I got a death threat.” (MAiD assessor/provider 26) “That was the longest death I’ve had [due to a complication from the forced transfer and lack of support from the objecting hospital] … when [the patient] finally died and the [family] had left the room, I just collapsed. I started bawling and … - I was trying to be quiet because this is not my grief, right. And I just started weeping and the paramedic came in and was like ‘It’s okay’. It was awful, just awful. I really felt I’d failed [the patient]. You know, I’d described the procedure and ‘it’ll be over in five minutes’. And then for this to happen, an ordeal like that, when if I could have requested [support] of the hospital this could have been avoided.” (MAiD assessor/provider 26) *Impact on professional relationships* “But there has been times where we’ve had to like not go there. Or we’ve had to really just like be battling it. And I knew going into it I was going into a hornet’s nest, and that happened. You know you’re meeting with hostility. No one’s going to help me. No one’s going to help me find what I need. No one’s going to give me the address to put on the death certificate. No one’s going to help me. So, I knew that and I could come prepared to be friendly, to be open, to be – you know, it’s all in the approach, right.” (MAiD assessor/provider 3) *Administrative burdens and lack of remuneration* “They were aware of the law. They were willing to proceed according to the letter of the law. But the palliative care nurse was told while they would not stop her from assisting it wouldn’t be done during her work hours, nor would it be part of her assigned work. She wouldn’t be paid for being there.” (Family caregiver 10)
6. Catalysts for change	*Description of changes over time* “…it was very different three years ago than it was now. So I did a lot of first provisions in hospice. They weren’t allowing them in hospice and they weren’t allowing the provision at all, and then they weren’t allowing admissions to do it. Over time it’s better and it’s evolved.” (MAiD assessor/provider 3) “In the beginning, yes that is definitely the way it was, that there were no assessments or let alone provisions happening in the hospitals.” (MAiD assessor/provider 5) *Positive experiences + normalisation over time* “…the underlying kind of drive comes from the public. It comes from people’s stories and it comes from publication of their stories and sharing of their narratives.” (MAiD team member 5) “Our laws changed because of patients. The patients challenge the system, change the law. The patients came to the community events that I spoke to. I couldn’t give rounds at the hospital that first month. I asked three or four different departments, can I come and give rounds on MAID. Nobody took me off on it, but I got three invitations in the community. Patients wanted to know. Patients took my information, went to their doctor’s office and said, hey, this exists, you don’t know about it, you find out about it, buddy, because I want – patients drive this change, and I think – and I watched it happen. Patients drove the change in the palliative care communities that were amenable to it. I know, several palliative care doctors that said, I respect what you do, I respect my patient’s choice, this is not something I’m going to ever do. A year later that doctor did an assessment for me. So it’s patient driven.” (MAiD assessor/provider 8) “I think they’ve come to some understanding that this is something that people who are Catholic wish to have, and it’s part of their healthcare, and they do recognise that it’s a legal right.” (MAiD team member 11) *Advocacy in response to negative impacts on patients* “… we vocally fought … and got the local press onside. We had a campaign. … [the institutions] wouldn’t have just dropped them [the restrictions] if we hadn’t fought that.” (MAiD assessor/provider 6) *Education and relationship building*
	“But I think the upfront work that the health authority did - so I have to say the [health authority] did a really good job of doing a lot of background institutional work and meeting with the nurses, the nurse practitioners, the long-term care workers. I mean very early on we had an educational session for all the physicians who worked in long-term care who were itinerant, you know who work in like three or four of the facilities, of saying ‘Here’s people who might be interested in this. Here’s what it looks like,’ and meeting with the nurses and social workers and care aides. Then with pretty well every institutional one that I did, even though we didn’t have the religious objective, I mean I sat down probably in about seven different long-term care facilities with the staff and said ‘Okay, well, Mrs Smith’ or Mrs Jones or whoever it is ‘has just died. Does anyone have any questions about how that process rolls out? How’s everyone feeling about it?’ You know, it’s difficult when they’re trying so hard to make that person’s life bearable and then they all of a sudden die. It’s like, no, you didn’t fail. It’s just that this person really had come to the end of their rope and they wanted to take some control back. So that was helpful. Because, unfortunately, a lot of the care workers worldwide are Filipino and Christian, right. I think in every single western, English-speaking country this is the reality. That’s hard for someone whose Christian values and sense of caring and duty are very, very strong. And I think it’s really worthwhile investing that time in the care workers.” (MAiD assessor/provider 12) “… a lot of that [allowing assessments on site] can be attributed to [team lead name] and our team, really. Because they worked super hard just making sure that there was education around what we were trying to do, education around responsibilities for objection, around transfer of care and what they mean to a critically ill, dying patient. So, I think that [team lead name] needs to be credited with all of that … [they] moved this along single-handedly.” (MAiD team member 1) *Institutional dynamics* “…the medical director, family doctor, palliative care doctor, and she told me they were the first hospice in the country to have a policy … She wrote the policy, so it was her initiative. She was not – that was not a requirement. I think it would be true of all the private facilities, long term care facilities, I doubt any of them have policies on medical assistance in dying.” (MAiD assessor/provider 11) *Regulatory mechanisms and leadership* “I would say it’s gotten a lot better. So it does sometimes come down to the directors and the medical leads, but I think that they have had a clear message from the health authority and from the province. It’s different if you are a member of the [British Columbia] Master Denominational Health Agreement, but if you are not and you are a publicly-funded facility then you do not have a legitimate argument to not support a resident who lives in your facility if they – you know, you are then blocking access to care.” (MAiD team member 5) “We said right at the outset that access to MAID was a *Charter* right for Canadian citizens and we were a public body. And, therefore, we would have no part of any of our facilities where MAID would not be permitted. It would happen where the patients are. We would have no death destinations and we’d have no opt out sites. So that got me a bit of heat early on… It turned out that was very much the best decision that we made right at the beginning because it set the atmosphere for the whole thing. We spent a lot of time educating.” (MAiD team member 8) “…there are some champions within the health authority… [who] really pushed it ahead. We thought it was going to go to a court battle and it didn’t, the health authority worked it out.” (MAiD assessor/provider 10) “…the health authority worked really hard to understand what they [the Catholic institution] wanted.” (MAiD team member 11)

### Theme 1. basis for institutional objection

There were two primary bases for institutional objections. First, objections based on religious values, which commonly arose in faith-based institutions (including hospitals, hospices, long-term care facilities, and home care providers). A physician noted:

*“Catholic hospitals and some of the Jewish hospitals will not allow it and some of the hospices that have religious affiliations won’t allow it. So, you don’t get to see patients in them.”* (MAiD assessor/provider 4)

The second basis for institutional objection was a particular philosophy of palliative care which arose in both faith-based and non-faith based palliative care settings (including hospices, and palliative care units within hospitals). For example:

*“In the beginning there were a lot of palliative care facilities that also just said, ‘well we won’t do that work.’ Non-faith based, just philosophically, ideologically.”* (MAiD assessor/provider 8)

*“There’s a large amount of overlap because many palliative care institutions are faith-based, especially hospices, because palliative care is sort of a ‘churchy’ speciality. I think palliative care has a role to play in that lack of access, in those difficulties of access as well.”* (MAiD assessor/provider 15)

Participants also reported that how an institution’s values shaped its position on MAiD was often determined by a key internal stakeholder, for example, the medical director of a palliative care unit, or chair of the board of directors. Staff in an institution were described as often being supportive of MAiD, despite the top-down decision. For example, a family caregiver commented:

*“…even though the majority of the people who worked at [institution] were intensely religious, either intensely Catholic or intensely Jewish … everybody supported [the patient’s] choice … It was specifically the board, and the chairman of the board, with them saying ‘No, we are too Jewish for that. We will not allow it’.”* (Family caregiver 28)

Similarly, a nurse practitioner noted:

*“…the people in the high positions, somebody has an opinion and becomes vocal and shuts it down for the entire facility.”* (MAiD assessor/provider 26)

Some participants provided examples in which the board’s position seemed driven by a desire to maintain the faith-based ethos of the facility, not because the board members necessarily agreed with this position, but rather to appease stakeholders, such as charitable donors, outside of the organization. For example:

*“Hospices in Ontario are funded mostly by charitable donations. … the board of [hospice name] would need to make a decision that we are … forgoing that gift and willing to pay for another piece of property in order to have the option of doing this.”* (MAiD assessor/provider 26)

Several participants highlighted that institutional objections in faith-based and palliative care hospitals, hospices, and aged care facilities were not universal. A physician noted:

*“For me institutionally, I haven’t had any issues. Our palliative care unit is very supportive of MAiD … the downtown one the same thing. … There’s no beef between palliative care and MAiD. Which when I first discovered that was a thing, it kind of blew my mind.”* (MAiD assessor/provider 20)

### Theme 2. scope of objection

Participants described a range of aspects of MAiD that institutions objected to. Some institutions would not provide any aspect of MAiD or allow it to occur onsite. For example:

*“…there is a care home downtown that I know I can’t go in and do an assessment there, I can’t go in and do a provision there, those patients need to come off the grounds of that building if I’m going to do assessments. I’ve done one in a café, I’ve done one in the park, done one on a park bench.”* (MAiD assessor/provider 8)

Other aspects that institutions objected to included: providing information; allowing request forms to be signed and/or witnessed onsite; transferring patient records to a MAiD assessor or provider; inserting the IV for MAiD provision; and enabling specialist consultations (e.g. a psychiatry consultation).

In contrast to institutions with blanket refusals, other institutions took less restrictive positions to MAiD. Some prohibited MAiD provision but allowed eligibility assessments. Participants also discussed hospices that would permit MAiD but would not allow patients to be admitted for this purpose. For example, a physician observed:

*“…they don’t admit people just for MAiD. So, if you were at home and wanted MAiD, they wouldn’t bring you in to get that, but they do have people who are there who request MAiD and have procedures there…”* (MAiD assessor/provider 11).

However, even in institutions with less restrictive positions, MAiD access could still be a problem. Some objecting institutions that allow MAiD assessments still refused to provide information about MAiD or tell patients it was an option. Participants reported this was related to the institutional position on MAiD, and in some cases also reflected individual conscientious objection of health professionals within the institution. For example:

*“I don’t think patients of [Catholic hospital] know that’s an option for them, because nobody’s telling them…there is an active access issue because providers in that institution are not telling people that it’s an option.”* (MAiD assessor/provider 11)

*“I’m a little surprised that the palliative care team that came to visit us didn’t tell us about MAiD. … That team very much didn’t bring up MAiD or that that was an option, and very much sort of proceeded in this, you’re-going-to-die-naturally-at-home way, was the sense that we got. So that was, in retrospect, a little surprising to me because that was definitely not what he wanted or intended.”* (Family caregiver 16)

Another barrier to access that participants attributed to institutional objection was the creation of additional logistics that slowed down or blocked a patient’s ability to access MAiD. A family caregiver described a long-term care facility that sought to have the patient use the institution’s form instead of the government one:

*“… that was one of these other brick walls they tried to throw up, is ‘No, we won’t accept her form. You have to use our form and our lawyers have to sign it,’ and we’re like “No, they don’t.” So they just kept coming up with nonsense to try and dissuade her…”* (Family caregiver 24b).

Many objecting institutions, particularly palliative care facilities, shifted from not allowing any aspect of MAiD to permitting assessments within a few years of MAiD becoming legal (discussed further in Theme 6). This change was attributed to several factors including institutions observing the impacts of forced transfers on patients, negative media attention, and the institution appreciating that an assessment is a conversation and believing “*that it’s only actually killing someone that’s not okay*” (MAiD assessor/provider 6).

Some participants highlighted that changes in institutional positions with respect to the scope of objections did not occur in a linear fashion, making them harder to navigate. For example:

*“… our hospital/hospice/nursing care home vacillated on what they allowed. First, they allowed nothing. Then they allowed a request form. Then they allowed assessments but only if we did it undercover. Then they wouldn’t allow forms. Then they would allow them again.”* (MAiD assessor/provider 24)

There was considerable institutional discretion in how MAiD requests were handled, and some participants emphasised that decisions about scope were often made on an ad hoc basis, resulting in the scope of objections between and within some institutions seeming inconsistent and arbitrary (Table 4).

### Theme 3. lack of transparency regarding institutional position

Participants described varying degrees of transparency about institutional positions. On one end of the spectrum were institutions with explicit policies against MAiD, which were clearly communicated to patients, health professionals, and care coordination teams, and were publicly available. For example:

*“They said, ‘We will not provide MAiD.’ It’s on their application to the hospice, the patients have to sign that they will not even discuss MAiD when they are admitted to a hospice.”* (MAiD assessor/provider 26)

In contrast, a family caregiver indicated that many institutions’ positions are not publicly promoted: “*I don’t think anyone on their website says we do or do not provide assisted dying*…” (Family caregiver 6).

A few participants emphasised that there was a lack of transparency about the extent to which institutions would facilitate, or require, MAiD transfers. A physician noted:

*“…if you ask some of the Catholic hospitals they will say, no, that they’re very compassionate and arrange these things [transfers for the purpose of MAiD]. My personal experience is that that’s not the case at all and that’s just nice talk. I mean I’ve recently had a couple of patients that have been in Catholic hospitals and there was no way we could get them moved to another hospital that allowed MAiD.”* (MAiD assessor/provider 4)

Similarly, a family caregiver described how it was only just prior to the planned MAiD provision, despite that patient having been approved for MAiD months earlier, when, *“*…*we found out that [the long-term care facility] would not allow her to have the procedure, her provision in her home”* (Family caregiver 28).

### Theme 4. impacts on patients

Institutional objections had several negative impacts on patients, across three broad domains: pain and other types of suffering relating to forced transfers; constrained choice regarding the patient’s end-of-life experience; and access being obstructed altogether.

#### Pain and other suffering related to forced transfers

The first major impact of institutional objection was pain and other suffering related to forced transfers out of objecting institutions. To sign forms and for MAiD assessments, patients were transferred to a variety of locations including other health care institutions, cafes, and public parks. For MAiD provision, patients were transferred home, to other institutions including hospitals and clinics, or to other locations such as funeral homes.

Forced transfers, when they occurred, had several consequences for patients. A physician described considerable pain experienced by a patient who was transferred out of a hospice for MAiD:

*“He had dozens of bone metastases from prostate cancer. I can’t imagine the agony of a bumpy ambulance ride for his death. It was just – [long pause] you know, we make oaths to do no harm, and I certainly felt that was a harm to this man.”* (MAiD assessor/provider 26)

Participants also described the emotional consequences forced transfers had on patients, including feeling stigmatised. A physician described a patient’s experience of stigma as being worse than the pain of the transfer:

*“… the ambulance ride was going to be painful for him. But he said … ‘Honestly, the hardest thing about this whole thing is this, having to come to a different hospital like I’m doing something wrong.’ So, it was like even in the absence of pain, in the absence of everything, just feeling like I’m doing something wrong.”* (MAiD assessor/provider 10)

Another emotional impact was imposing additional logistical roadblocks (which participants perceived were due to the institution’s objection to MAiD) which negatively impacted the patient’s MAiD experience:

Family caregiver 1: *“There’s enough pain as it is. To then throw up these roadblocks on top of it is just cold….”*

Family caregiver 2: *“…that’s harm that can’t ever be undone.”* (Family caregivers 24a and 24b)

#### Constrained choice and other impacts on the person’s end-of-life experience

A second major impact of institutional objection on patients was constrained choice and other negative impacts on the patient’s end-of-life experience. For example, some patients were forced to choose between MAiD and being admitted to hospice. A nurse practitioner noted:

*“…in this [regional] community…it’s a huge barrier. We can’t go into hospice. In fact, hospice tells patients when they’re interviewing them that if they are considering MAiD they will not be allowed to come into hospice. … some families cannot cope with palliative care at home.”* (MAiD assessor/provider 23)

Another example of constrained choice was that some patients were compelled to access MAiD in less-than-ideal locations. Participants described developing suboptimal solutions when a person did not have a residence to be transferred to, including transfers to an abortion clinic, HIV hospital, a boardroom, and a basement. A physician described struggling to find a place in a rural area for a patient who did not want to have MAiD at home for the sake of his young children:

*“I am worried about the one gentleman … I’m not sure where he’ll go. I heard that provincial parks will allow MAiD. So I was going to contact … [name of provincial park] and see if we could go there, but it seems bizarre. I’d be willing to bring him to my backyard, you know. It’s strange not having any place to offer these people when they could legally probably die in the Tim Hortons [coffee and donut shop] parking lot, you know, or the middle of the street, but not in a hospital or a hospice. It makes no sense to me. I find it immoral.”* (MAiD assessor/provider 26)

#### Obstructed access

A third impact on patients was that access to MAiD was at times precluded, for a variety of reasons. For some, it was simply too hard to pursue MAiD in the context of the institution’s objection. A physician commented: *“the persistence required to even get an assessment or have a conversation … about MAiD in a Catholic institution … is enormous”* (MAiD assessor/provider 15).

In other cases, patients were too sick to be transferred, some experienced a medical complication and died during the transfer, and some lost capacity due to medication needed to make the patient comfortable during the transfer. For other patients, MAiD access was compromised because they did not want to leave the facility where they had been living and where they knew the staff. A physician commented:

*“I have had a number of patients that I assessed and were approved for MAiD in the hospital, but they said I’ve been living in this palliative care ward now for weeks and in some cases months, these are my family, I don’t want to leave them. I don’t want to go where there are strangers, where people don’t know me …”* (MAiD assessor/provider 5).

#### Mitigating factors

Participants described several factors which mitigated these negative impacts on patients, including patient assertiveness. For example, *“she was bound and determined this was what she wanted”* (Family caregiver 24b). Also significant were family caregivers who advocated for the patient and were willing and able to facilitate the patient’s choice. For example:

*“… they [the long-term care facility] actually told us she wasn’t allowed to access assisted dying there. And we said ‘That’s absolutely wrong. This is her home. She is legally – you are legally obliged to allow her to access it there.’ So again we had to fight that fight.”* (Family caregiver 24a)

*“…when that time came when we were scrambling to get a bed, because there’s not very many spaces available, we were told by a friend who had connections with hospice who said ‘If your intention is to have MAiD, don’t mention that in your hospice intake.’ … in general we were told, ‘Just keep that quiet just in case you come across somebody who’s not supportive. You can’t say that your intention is to enter hospice to have MAiD.’ So we didn’t say anything. Once we got there, then the conversations were okay…”*

(Family caregiver 6).

MAiD assessors/providers and MAiD team members also played an important role in mitigating harm, using their knowledge of where to steer patients who are considering MAiD. For example:

*“I always make sure to say, ‘But if you even think you might at some point in the future want an assisted death, do not go here and do not go there. Go here.’”* (MAiD assessor/provider 15).

MAiD assessors/providers also described advocating considerably for patients, contacting hospital administrators and the media in egregious cases. Family caregivers spoke about the incredible personal dedication of clinicians, describing one physician as someone *“…who would move mountains to serve somebody however and wherever they need to be”* (Family caregiver 28). Some participants discussed assessors and witnesses who entered facilities posing as a visitor or family member for the purpose of signing forms or doing an assessment (though others expressed discomfort with this practice). For example:

*“…we used all sorts of other tactics to get around it but, at the end of the day, they insist that patients obviously leave the facility to have MAiD.’”* (MAiD assessor/provider 27)

Another mitigating factor was pre-existing care pathways set up to navigate the objection. One example was a partnership between a long-term care facility that refused to provide MAiD and a MAiD coordination team at a hospital cluster to facilitate the MAiD process: *“…you had someone who you could discuss [MAiD] with, without having to involve the long-term care home”* (Family caregiver 12). Another example mentioned by several participants was a faith-based hospital with a dedicated separate area attached to it where MAiD provision was permitted.

### Theme 5. impacts on health practitioners

Participants highlighted three main impacts on health professionals caused by institutional objections: emotional impacts; impacts on professional relationships; and administrative and workload impacts.

#### Emotional impacts

First, participants described significant emotional impacts on health practitioners from dealing with institutional objections, including frustration, anger and disgust, moral distress, and feeling stigmatised. A physician described feeling as if they had “failed” the patient who had a prolonged death after a forced transfer. Another physician discussed feeling outraged that their patient had experienced a series of forced transfers for assessments and provision:

*“I was outraged by this one and I blew up. … Because we’re supposed to be all about patients and how is this possible?”* (MAiD assessor/provider 17)

Another emotional impact on health professionals was stress from being subject to threats including complaints to various authorities, such as their professional regulatory body (College) and the police, and in one case, a death threat (Table 4). Participants indicated that although stressful, none of the complaints to the various authorities eventuated in sanctions.

#### Professional impacts

Second, participants discussed impacts on professional relationships caused by institutional objections, including employer-employee relationships. A key subtheme was situations involving a perceived conflict between the institution’s position and the views of staff. A number of participants described institutions with staff that were supportive of MAiD who did not agree with the institution’s position. For example:

*“…most of the clinicians that I know who work in Catholic institutions would gladly provide MAiD, it’s just that they’re not empowered to*.*”* (MAiD assessor/provider 15)

As mentioned in Theme 1, the institutional position was often attributed to opposition by a key stakeholder such as a medical director or board of directors. Another professional impact was tensions with other health professionals, such as experiencing hostility and a lack of assistance in a facility from employees who appeared to share the institutional position.

#### Administrative and workload burdens

Third, health practitioners took on additional administrative and other workload burdens to navigate institutional objections. Some staff were allowed to participate in MAiD, but only in their own time and without institutional support. For example, a palliative care nurse whose employer did not support MAiD was informed they could participate but only outside work hours, and therefore unremunerated. Several participants describing having to scramble to find locations for their patients to receive MAiD assessments and/or provision. A nurse practitioner commented:

*“The fact that she actually ended up having MAiD how she wanted in the community was a shit ton of work on my part and the connections that I had. I’m not saying that to toot my own horn. It literally was she just happened to be seen by the right person, and that’s sad.”* (MAiD assessor/provider 1)

A MAiD assessor/provider in a rural area recounted that this additional workload was considerable because none of the local hospitals, hospices, or long-term care facilities would allow MAiD. The physician commented:

*“… this is taking a lot of my time and mental head space I’d rather be spending on my kids than writing letters to CEOs of hospitals.”* (MAiD assessor/provider 26)

### Theme 6. catalysts for institutional change

A final theme was catalysts for institutional change. As noted in Theme 2, participants highlighted that in some (but not all) settings, institutional objections relaxed somewhat in the six years since Bill C-14 was passed, reducing negative impacts on patients.

*“Those Catholic hospitals that at the beginning would never let you in the door, now they let you in the door to assess people.”* (MAiD assessor/provider 21)

*“I would say that 90% of [faith-based institutions in the province] are very, very supportive and they have now moved to allowing assessments but not provision. So there’s less and less feedback from patients and family about adversity within that setting.”* (MAiD team member 5)

The position in non-faith based palliative care settings was reported to have changed more than in faith-based palliative care settings. A physician noted:

*“… some of those facilities have now moved a little bit more towards the middle or even allow assessments and provisions to happen. Each of those facilities have found their level. … the whole spectrum exists in a hospice or palliative care facility. So there’s been a lot of movement in that community. Not so much in the religious based ones, though.”* (MAiD assessor/provider 8)

#### Greater acceptance of MAiD over time

Participants attributed changes in institutional positions to a variety of catalysts. One prominent catalyst was greater acceptance of MAiD over time, due to positive patient experiences, growing comfort in the medical community, and destigmatization of MAiD. A physician commented:

*“I think they [decision-makers in an objecting faith-based hospice] … were accustomed to seeing people suffer quite badly … they saw how humane MAiD was and how grateful the patients and the families were.”* (MAiD assessor/provider 4)

Likewise, a physician in Ontario described how institutional change was prompted by individual patients seeking MAiD as an end-of-life choice:

*“Those Catholic hospitals … now they let you in the door to assess people. So it’s changing because they recognise that this has become … a standard of practice and they need to get on board and give people options.”* (MAiD assessor/provider 21)

Another related factor was growing comfort with MAiD in the medical community. This was driven in part by clinicians witnessing MAiD assessments and observing peers they respected engage in MAiD work. For example:

*“Once they [other clinicians] experience it and they see how gentle and … the gift that you give a family with the provision and that opportunity, I don’t know, it’s hard to stay too closed about it for too long, at least in my experience.”* (MAiD assessor/provider 3)

*“Some of the palliative care doctors that were very opposed at the beginning are now the staunchest allies.”* (MAiD assessor/provider 4)

Another participant noted that, as in other areas of social change, broader societal acceptance of MAiD has grown with time and experience, reducing stigma:

*“As time has gone by and as society has come to understand – much in the way that when medical marijuana was legalized, society did not end. … They saw the same thing for abortion. … They saw the same thing for MAiD and people are understanding now that society didn’t end … time is a big thing. … Acceptance of the procedure and getting a sense that it is actually tremendously well-regulated … that there’s a process, that there’s due diligence, that there’s two assessments, that the person has to meet criteria, that this is overseen is an important thing as well. And then word of mouth, right … you don’t have to go too far before you meet someone who [has a relative that had MAiD] … it’s less taboo.”* (MAiD team member 13)

#### Advocacy to promote patient access and address the harms of institutional objection

A second catalyst for change was advocacy to address the negative effects of institutional objection on patients. In addition to mitigating harm on an individual patient (discussed in Theme 4), advocacy for individual patients also contributed to broader institutional change. Advocacy was undertaken by patients, family caregivers, MAiD assessors/providers, and organizations such as Dying with Dignity Canada and CAMAP. A physician commented on the important role of on-the-ground advocacy by patients and clinicians:

*“*… *between the patients driving it on one end and the clinicians who are in the community itself driving it – that’s what causes change in this country, those two forces.”* (MAiD assessor/provider 8)

Advocacy was particularly effective when amplified by media reports. For example, a physician recounted how media attention on a particular case impacted an institution’s discretion:

*“… I talked to the family, and they said “Oh, yes, we’re going to the press” … we went to the press that this poor man had to be transported … this was outrageous and awful. And guess what, ever since then every single patient at that facility has been assessed as requiring an in-hospital assessment [as opposed to being transferred off site].”* (MAiD assessor/provider 22)

#### Education and relationship building

A third catalyst was education and relationship building with objecting institutions. Participants highlighted proactive work by MAiD teams in some health authorities, who met with institutional decision-makers and staff. For example:

*“… I think the upfront work that the health authority did … [they] did a really good job of doing a lot of background institutional work and meeting with the nurses, the nurse practitioners, the long-term care workers.”* (MAiD assessor/provider 12)

Individual MAiD assessors/providers also described participating in education and relationship building, through speaking with staff in objecting institutions about what the MAiD process entailed and the impacts of transfers.

#### Institutional dynamics

Institutional dynamics were another catalyst for institutional change. Participants described how greater acceptance over time by clinicians within an institution contributed to changes in institutional policy. For example, a physician described how attitudes changed towards MAiD in a hospice that allowed external assessors to provide MAiD assessments:

*“I could go in there, do an assessment, speak to a patient, leave. I would come in almost like a specialist. … I followed the rules happily, respected each other, did the work. It went from that, which it stayed at for a couple of years. Then of course the inevitable, which happens all the time. The palliative care doctors … they’re the most patient centred doctors on the planet, they are very attached to some of their patients, they’ve known them for a while, they’re quite connected, they work hard with them, and those patients start begging them for help and they start asking them for assisted deaths. Some of those clinicians started feeling like … this is my patient, I can help this patient. So they started to want to – there is that one case, like this one I’m going to support. So they’d asked me and I showed them how to do the assessment form then they would do it. Then they would only do it maybe once or twice a year, and then all of a sudden they’re like, well I can do this, this patient of ours, I can do it. All of a sudden we’ve got now maybe half of them are willing to do assessments.”* (MAiD assessor/provider 8)

Participants also cited leadership by dedicated individuals and support from staff or clinicians at various levels within an institution who supported MAiD access. Participants discussed champions in organisations, such as medical directors, who developed policy to support MAiD, and decisions by boards or CEOs to support MAiD (or establish processes to foster patient-centred care). For example:

*“I met with the CEO of the hospital. … I had [CEO] behind me, he said ‘Yeah, this is – let me be clear, this is happening at this hospital, and it happens the way it needs to happen and that’s all there is to it.’ Obviously, there were political pieces that had to be done very carefully…”* (MAiD assessor/provider 9).

#### Regulatory mechanisms and health system structures

Finally, some participants cited the role of regulation and health system structures in fostering institutional change. This included government policy and agreements, and policy set by MAiD teams in some areas. For example, a MAiD team member noted:

*“*… *this health authority and [names of key leads] and the director at the time were very clear about access to care.”* (MAiD team member 5)

Another MAiD team member noted the importance of the provincial regulatory framework in British Columbia, which required long term and residential aged care facilities to provide information about MAiD:

*“The provincial government did say quite clearly that information is to be made easily accessible to all residents in these facilities. So they’re not allowed to restrict information access.”* (MAiD team member 8)

Similarly, participants from Nova Scotia described how an objecting hospital’s position changed through the negotiation of a separate space attached to the hospital that persons could use for MAiD. This was achieved through advocacy by several key regulatory stakeholders.

Participants perceived that the absence of more formal regulatory mechanisms and top-down decision-making in fostering system change resulted in insufficient protection for patients from institutional objections. Despite descriptions of strong support by higher authorities in some locations, in others, participants felt that the regulatory environment still lacked sufficient protection for patients. For example:

*“I wish the Ontario government would say ‘No, you will all provide’ … You get a dollar of our money, then you will provide all services…”* (MAiD assessor/provider 26).

Similarly, a participant from British Columbia noted that government agreements would need to be amended to make changes:

*“… in some provinces like the one that I’m in, there is actually a contractual agreement from the ‘90s that allows faith-based facilities to dictate what happens on the premise. So we probably can’t break that contract or it needs to be re-looked at.”* (MAiD assessor/provider 8)

However, some participants acknowledged that this and other government-led change was highly political: *“I can understand why the politicians don’t want to touch it…”* (MAiD team member 8).

## Discussion

There are relatively few studies focused on stakeholders’ experiences of institutional objection to MAiD in Canada, [8] and internationally [26]. In our broader study on MAiD decision-making, institutional objection was a frequently cited issue, with 27/32 MAiD assessors and providers, 9/11 MAiD team members, and 9/31 family caregivers having direct experiences of it. This study identified six themes related to institutional objection, drawing on reports of family caregivers, MAiD assessors and providers, and members of MAiD teams in British Columbia, Ontario, and Nova Scotia. Several themes resonate with the small body of existing literature, particularly the bases for institutional objection and impacts of forced transfers on patients. However, this research also provides novel insights, including factors leading to improved patient access to MAiD in response to institutional objections. Another unique finding is how some institutional objections have eased in the six years since MAiD was legalized federally in Canada, and the catalysts for that change. Despite these positive changes, participants reported institutional objection remains a significant problem in some settings, with a number of ongoing challenges. This study provides lessons for other jurisdictions, such as Australia, New Zealand, Spain, and various US states, where MAiD laws are more recent and are in the process of being implemented.

### Patient harm demonstrates need for supports to access MAiD

Institutional objection spans a variety of practices and causes a range of harms to patients and their families. Consistent with other research, [8, 26] transferring a patient for MAiD assessments and provision was reported to cause pain and other emotional and psychosocial impacts. In addition to assessments and provisions, this study found a wider range of practices institutions refuse to engage with, including signing and witnessing request forms, IV insertion, and referrals for specialist consultations. Suffering due to institutional objection is therefore not only attributable to forced transfers, but also to less visible sources of stress including stigma, logistics, and administrative burdens. Institutional objections also constrained choice about how, when and where MAiD could be accessed, and disrupted existing therapeutic relationships, interfering with key parts of quality care [38].

This study identified several factors that mitigated negative impacts on patients and practitioners (discussed in Themes 4 and 5), which echo findings in previous research in Canada, [8] and Victoria, Australia [26]. Access to MAiD in an objecting institution often depends on individuals who are willing and able to drive the process and, in some cases, challenge the institutional position. This requires considerable effort and tenacity on the part of patients, family caregivers, and health professionals.

However, despite such efforts, a relative power asymmetry remains between individuals and institutions which can impede access to MAiD. For patients this is exacerbated given they are suffering from a grievous and irremediable condition. Further, not all patients have the ability, energy, supports, or resources to advocate for themselves. Additionally, patients may often have no choice about where they are treated. An objecting institution may be the closest or only health facility in the patient’s area (a particular problem in rural settings) and may be the only facility to provide specialized care, such as palliative care [20, 22]. In some cases, patients may not be aware of the institution’s position and how this may constrain their choices [39, 40]. Power asymmetries can also exist for health professionals, particularly if they are employed by an objecting institution or if they work in a region where the only hospital is a faith-based institution that will not grant them privileges, impeding their ability to advocate for patients. Our findings, therefore, suggest a need for regulatory structures and MAiD programs that support patient access in the face of institutional objections.

### The need to increase transparency and clarity regarding MAiD access in objecting institutions

A factor that compounded impacts on patients and created challenges for health professionals was that institutional decision-making often lacked transparency and was subject to considerable discretion. At times, participants reported it was unclear what the institutional policy was, whether discretion would be exercised in favour of the patient to access MAiD, and how to navigate around barriers. This is consistent with literature demonstrating a lack of transparency in the positions of faith-based institutions, [39, 40] and uncertainty about who is safe to trust within those institutions [25]. “Pathway ambiguity” (i.e. a lack of clarity around care processes and challenges in care coordination) is problematic in MAiD in general, [25, 38, 41] and our findings suggest institutional objections contribute to this problem.

While robust care coordination can mitigate pathway ambiguity, the variable expressions of institutional objections may pose challenges to the effective coordination of the MAiD process. Other research has demonstrated that poorly coordinated care can be disruptive to the patient, the family, and the clinical team [38]. Our study highlights the benefit of a proactive approach to MAiD teams making contact with institutions and their staff and engaging in relationship building and education. However, our results also emphasize the success of this approach is highly dependent on local setting and the willingness of higher-level authorities to set clear policy promoting MAiD access. Some geographic areas do not have robust care coordination processes or willing institutions. A lack of transparency impedes patients’ ability to make choices about their care. Ideally, institutions should be required to disclose their position transparently and proactively, and systems should promote consistent and clear decision-making.

### Diversity within institutions suggests support needed for conscientious participants

This study identified two primary bases for institutional objections to MAiD: religious values and a philosophy of palliative care that sees palliative care as incompatible with MAiD. Both are well documented bases for institutional objection in the empirical [8, 24–26, 29, 45] and theoretical literature [3, 4, 7]. A further finding of this study was that participants perceived that institutions’ objections to MAiD were often driven by top-down decisions, rather than a view universally shared by staff.

In other aspects of healthcare, such as reproductive medicine and contraception in faith-based hospitals, a lack of congruence between an institution’s position and staff views contributes to professional conflicts and moral distress [42, 43]. While evidence on this issue in the MAiD context (both in Canada and internationally) is still emerging, the same challenges appear to exist. In a study of physicians and nurse practitioners, Brown et al. (2021) found some participants were frustrated by institutional objection, while others were comforted by the institution’s stance [44]. Research on perspectives of palliative care unit and hospice staff suggests some palliative care practitioners view MAiD as a departure from “usual” practice, [45, 46] while others believe patients should have a right to access MAiD in hospice [45].

Our findings confirm that some health professionals involved in transfers from objecting institutions experience moral distress by being compelled to be involved as they see these transfers as not in patients’ interests. Participants also reported a diversity of views from health professionals employed by objecting institutions, including those who conscientiously object to MAiD and those who would want to assess or provide (or otherwise support the MAiD process), but for the institutional position. Practical and professional supports are needed to support this diversity of views. Difficult professional dynamics due to conflicting views within an institution have emotional consequences for providers, and can affect provider willingness to participate in MAiD [27, 28]. While there is substantial literature on support for conscientious objectors to MAiD, our findings underscore the need for laws, policies, and practices to go beyond just protections for health professionals who conscientiously object and also extend to conscientious participants [27].

### Implications for system change: the value of a multi-pronged approach underpinned by regulation

A new finding of this research is that some of the problems associated with institutional objections in Canada have improved over time, at least in some places. Participants reported increased acceptance of MAiD from previously objecting institutions, and a wide range of catalysts that contributed to changes in institutional positions. The catalysts we identified reflect both bottom-up forces, such as patient demand for MAiD and voluntary efforts by clinicians to effect system-wide change, and top-down ones, including regulatory architecture and strong policy positions from local authorities. These findings suggest a multi-pronged approach contributes to improved access and patient and provider wellbeing.

Bottom-up catalysts were critical in effecting change in the first six years since MAiD became legal. Just as family perspectives on MAiD may become more favourable with direct experience, [47] this study suggests that so can health professionals’ and institutions’. The impact of observing positive patient experiences with MAiD, examples of integration between MAiD and palliative care, and patient demand for the option, led some institutions to soften their positions. Additionally, the influence of clinical leaders and peers who support MAiD led to greater acceptance and engagement in MAiD by individual clinicians, which in turn contributed to changes in institutional culture.

However, the persistence of institutional objections within faith-based institutions (relative to the changes observed in some secular palliative care settings) suggests that institutional objections rooted in religious values or ideology may be less amenable to change. This is another factor that suggests a stronger regulatory response may be needed, which reduces the need for bottom-up advocacy by patients, family members and health practitioners. Advocacy by very unwell patients and their families in response to a roadblock caused by institutional objection is a significant burden, and many patients may be simply unable to advocate due to factors including how unwell they are. The absence of top-down regulation may impose a significant burden on clinicians to undertake advocacy and negotiate patient access. Given that the model of assisted dying in Canada relies on clinician involvement to facilitate patient access, reducing burdens on clinicians is also important in ensuring provider sustainability and, in turn, patient access [27, 48].

While many changes happened “organically” over time, our findings suggest top-down policies and regulatory mechanisms are critical in supporting patient access in response to institutional objection. Locations where MAiD assessors and providers and MAiD team members indicated they had encountered few issues with institutional objections were ones where the health authority or medical director of an institution had proactively established a strong position supporting access. Further, while our study design cannot provide insights into prevalence of institutional objection, participants in Ontario generally reported more widespread challenges than in British Columbia and Nova Scotia. There is considerable variation in MAiD regulation and service delivery across Canada, both between and within provinces and territories. As noted in the introduction, while the MAiD law is set out in the federal *Criminal Code*, healthcare in Canada is implemented by provinces and territories with Nova Scotia, and to some extent, British Columbia implementing stronger regulatory support for patient access to MAiD. The Nova Scotia Health Authority has required all publicly-funded facilities to allow access to MAiD [12]. Participants reported that British Columbia has facilitated access through provincial policy requiring the provision of information and, requiring non-faith-based institutions receiving greater than 50% of their funding from the government to allow access to MAiD (though the provincial MAiD policy and the Master Agreement between the province and the Denominational Health Care Facilities Association allow publicly-funded faith-based institutions to refuse to allow the provision of MAiD within their walls) [15]. Obviously, where there is legislation or policy requiring access, access is less impeded.

This may mean that a stronger top-down regulatory response to institutional objection is needed in areas where problems with MAiD access remain an issue. Although participants described significant improvements in patient access in many geographic areas, institutional objection remained a problem in many places, particularly rural and remote regions where no local institutions supported MAiD. However, even where more formal regulatory instruments and top-down policy exists, our findings indicate some objecting institutions introduce more subtle barriers to access, such as adding bureaucratic roadblocks not required by law or provincial policy. Bottom-up reporting of experiences by patients, families, and clinicians will remain important in highlighting and overcoming more surreptitious forms of institutional objection impeding access to ensure genuine non-obstruction and facilitate patient access.

### Limitations

A strength of this study is it includes the perspectives of multiple family caregivers (reporting on the experience of patients they were supporting as well as their own experiences), MAiD assessors and providers, and MAiD team members. Drawing on these three cohorts across three provinces provides a robust basis to identify both individual and systems issues. A potential limitation is the perception of family caregivers may differ from those of patients, and can be influenced by grief, bereavement and their relationship with the patient [35, 49]. Although we attempted to recruit patients seeking MAiD, this is a difficult cohort to reach. While family members have been demonstrated to reliably report on the quality of end-of-life care and on observable symptoms, [50] more research involving direct patient voices is needed.

An additional potential limitation is that our family caregiver sample was predominantly female. Further research on how gender may play a role in patient advocacy may provide additional insight into the dynamics between institutions, patients, and families. Another limitation is that all family caregiver interviews which reported patients experiencing an institutional objection ultimately involved the patient accessing MAiD. While MAiD assessors and providers also commented on cases when patients did not access MAiD due to institutional objection, further research on family or patient perspectives when patients are prevented from accessing MAiD is needed.

Our sample may also be more supportive of MAiD and more opposed to institutional objection given recruitment involved study advertisements circulated by Dying with Dignity Canada (a key patient education and interest group) and CAMAP (the national professional organization for MAiD). However, subsequent purposive sampling for diversity and the inclusion of members of MAiD teams may have helped to ensure a range of views were included in the sample. Even so, more research is also needed from the perspective of objecting institutions, and from healthcare professionals who work in them (including those with a conscientious objection to MAiD). Our study suggests that the ethos for an institution is often determined by a top-down decision, but how this operates and may change over time warrants further investigation. Similarly, more insight into how institutions develop and apply their MAiD policies, and how this affects patients, family, and staff in the institution, is warranted.

Finally, since the regulatory environment regarding institutional objection and implementation of MAiD into the healthcare system varies by province and territory, additional research exploring the impacts of institutional objection in other Canadian provinces and territories may reveal different or additional experiences and catalysts for change. More data on prevalence of institutional objections and patient transfers, and the impact of geography on patient access, would also be valuable.

## Conclusion

The ethical justifiability of institutional objection is contested, and this study raises questions about how best to address harms caused by institutional objections. These findings shed light not only on Canadian MAiD regulation and practice but are also relevant to other jurisdictions which have legalized MAiD or are implementing it or considering doing so. Should objections be regulated by the state or left to individual institutions to negotiate? If the state chooses to regulate institutional objection, should this be achieved through law or policy or some other mechanism, and what model of regulation is appropriate (e.g. permitting institutional objection wholesale, not allowing it or some type of reasonable accommodation model that aims to balance patient and institutional interests) [3]? The wide range of harms identified, both to patients and practitioners, suggest that at least some limits to institutional discretion are warranted and that top-down regulatory involvement may be the best way to facilitate patient access to this lawful end-of-life choice.

### Electronic supplementary material

Below is the link to the electronic supplementary material.

Supplementary Material 1

Supplementary Material 2

Supplementary Material 3

Supplementary Material 4

## Data Availability

The interview guides are available in Additional Files 1, 2, 3, and 4. Due to confidentiality undertakings given to research participants as a requirement of the study’s ethical approval, the data generated and analyzed in this study are not publicly available. Requests to discuss this should be directed to the corresponding author.

## Abbreviations

MAiD: Medical assistance in dying

## Footnotes

1 MAiD was legalized federally in 2016 when the Canadian government introduced Bill C-14, An Act to Amend the Criminal Code and to Make Related Amendments to Other Acts (Medical Assistance in Dying), 1st Sess, 42nd Parl, 2016 to respond to the Supreme Court of Canada’s decision in *Carter v Canada (Attorney General)*, 2015 SCC 5. However, provincial legislation permitting MAiD was first enacted in Québec which commenced operation in 2015: *Act Respecting End-of-Life Care*, RSQ c S-32.0001.

2 The most recent Health Canada annual report states that there were 10,064 MAID provisions in 2021 and “fewer than 7” (0.0007%) were due to self-administration (a trend consistent with previous years). See: Health Canada. Third annual report on medical assistance in dying in Canada 2021. 2022. https://www.canada.ca/en/health-canada/services/medical-assistance-dying/annual-report-2021.html. Accessed 17 Apr 2023.

3 In Canada, criminal law is the responsibility of the federal government, while the administration and delivery of health care is the responsibility of the provinces and territories: *Constitution Act 1867*. The federal *Criminal Code of Canada*, RSC 1985 c. C-46 (“*Criminal Code”*) contained prohibitions on MAiD, which were amended by Bill C-14, An Act to Amend the Criminal Code and to Make Related Amendments to Other Acts (Medical Assistance in Dying), 1st Sess, 42nd Parl, 2016 and subsequently by Bill C-7, An Act to Amend the Criminal Code (Medical Assistance in Dying), SC 2021, c. 2. While the core legal parameters for MAiD are set out in the *Criminal Code*, how MAiD is administered and delivered is up to each provincial and territorial government. For more information see: Downie J, Scallion K. The path from Rodriguez to Bill C-14 and beyond: lessons about MAiD law reform from Canada. In: White BP, Willmott L, editors. International perspectives on end-of-life law reform: politics, persuasion and persistence. Cambridge: Cambridge University Press; 2021. p. 17–39.

4 The sole patient in the total sample was living in the community and did not experience institutional objection but described engaging in advocacy to combat it.

5 Note that all totals reflect that 5 participants had overlapping roles as described in the results.
