# Supplementary material for: A qualitative study of experiences of institutional objection to medical assistance in dying in Canada: ongoing challenges and catalysts for change
Source: BMC Med Ethics. 2023 Sep 21;24:71. doi: 10.1186/s12910-023-00950-9 (PMC10512474; doi:10.1186/s12910-023-00950-9)
Supplement: Supplementary file 1 — Supplementary Material 1 [file 12910_2023_950_MOESM1_ESM.pdf]

OVERVIEW OF INTERVIEW GUIDE

- FAMILIES -

Introduction

- Introduce interviewer(s) and study.
- Thank you for being able to help with this research. Before we go into detail, can we just first deal with the administrative side of things?
- [Zoom recording, consent, and confidentiality discussion].
- You may feel that some of the questions I ask are stressful or upsetting. If you do not want to answer any of these questions, please just say so. There are no right and wrong answers; we are simply interested in your views.
- Do you have any questions for me before we start the interview?

Overview of individual seeking MAiD

Before we go on to talk about your experiences of MAiD and how the process went for your family member, would it be possible to give us a short picture of your family member? We will talk about this some more – but just a nutshell, even a minute or two, just to help us get to know [family member] first.

Remaining interview approach

In terms of the rest of our discussion, I know we have a lot to cover and I want to make sure we can capture it all. To start let's step through the MAiD process in order so you can comment on each part of the process. I will then finish with some more general and open-ended questions which will also provide a chance for you to tell me other things. Is it OK if we proceed that way and perhaps if am a bit directive to make sure we understand your experience?

First discussion of MAiD

- What was the extent of your exposure to MAiD (if any) prior to your experience with your family member?
- When did your family member first become aware of MAiD and that it might be possible? How did this topic come up (e.g. sources of information)?
    - Prompts: Who did they talk to about this? Were you involved in these conversations?
    - Why did you/they go down this path of seeking information about MAiD? (Motivation, what they wanted).

  - If individual or family member was involved in prior MAiD advocacy or reform, ask further information about nature of involvement and motivations for involvement in this work.
- Was it easy to get information about MAiD or were there barriers?
  - Prompt: Where did you/they go for information?
- When did they first talk about MAiD with a physician or nurse practitioner (or other health professional/care coordinator)? Who raised it? How did the health professional respond?
  - Prompt: Were there any barriers or concerns with their physician, nurse practitioner, or other health professionals?

Assessment process

- Can you tell us when your family member sought MAiD [and which track they were on (if post March 2021)]?
- What was their experience of requesting and being assessed for MAiD? How did the process start?
  - Prompts: Were they able to easily find a physician/nurse practitioner to guide them through the process? Were they connected to a care coordinator or other service?
  - Can you recall whether they had to make a written request to a physician or nurse practitioner to start the process? How did this work?
  - Were there any difficulties in getting a witness (or two if pre-March 2021) for this written request?
  - Who located the health professionals to do the assessments? Were there any difficulties in finding two independent physicians/nurse practitioners to confirm they were eligible?
  - Were there any difficulties with eligibility criteria? [Particularly ask about specific eligibility criteria if mentioned as an issue].
    - [Note after March 2021, reasonably foreseeable natural death is no longer an eligibility criterion but does determine whether a person is Track 1 or Track 2].
  - Do you recall how long the process took from the written request to when they were found eligible for MAiD?
- General prompts:
  - Did you/they need to contact any care coordination services?
  - Was telehealth ever used for MAiD consultations?
  - Were there issues with travel to access health professionals for assessments or for the provision of MAiD?
  - Where were they living when seeking MAiD? Did their facility facilitate access to MAiD or was it a barrier to access? How? Was this explicit or implicit? [e.g. institutional objection].

- What did they think about the waiting period? [Note: prior to March 2021, 10-day waiting period and after March 2021, 90-day period for Track 2 (when natural death not reasonably foreseeable), but potential to be shortened if loss of capacity imminent].
  - [If Track 2 (post March 2021, natural death is not reasonably foreseeable) and neither assessor/providers have expertise in the illness/condition]: Were there any difficulties finding an independent practitioner with expertise in the condition causing the person's suffering? [Note this would often be arranged by clinicians/care coordinators].
- Overall, looking back at the request and assessment process, was the process straightforward? Challenging? Why?
  - Prompts: What parts of the process worked well? What parts (if any) needed improvement?

Prescription of medication and provision of MAiD for eligible MAiD patients

<u>If provider administered:</u>

- Did your family member have provider-administered MAiD? Was there any discussion of self-administration as an alternative? If so, what were you/they told and what impression were you left with about it?
- Can you walk us through what happened from when the provider arrived until when your family member passed away?
  - Prompts: How long did the process take from when they started seeking MAiD to when your family member had chosen a date to receive MAiD?
  - The choice about whether or not to have MAiD and when to have it – that was a choice. What was guiding/guided them about the decision to have MAiD when they did?
  - Looking back, what aspects of the process for MAiD provision work well and what needs improvement?
  - [If after March 2021 and Track 1 (natural death reasonably foreseeable)] Did they consider a final consent waiver?
  - What was their experience of this (in terms of making the written arrangement and/or in the arrangement being followed)?
  - [If not at home] Did their facility facilitate the MAiD provision or did the facility create any barriers? How? Explicit or implicit? (E.g. institutional objection).
  - What was the process after your family member's death?

<u>If self-administered:</u>

- How was the process of obtaining and using the medication? Can you walk us through what happened from when your family member obtained the medication until when they passed away?

    - Prompts: any involvement in preparing the medication, experience of person taking it, experience with pharmacists.
    - Once they got the medication, how did they feel about that?
    - The choice about whether or not to take it, and if they did, when to take it – that was a choice. What was guiding/guided them about the decision to take the medication and its timing?
    - Looking back, what aspects of the process for getting the medication/using MAiD medication work well and what needs improvement?
    - [If not at home] Did their facility facilitate access or was it a barrier? How? Explicit or implicit? (E.g. institutional objection).
    - Prompts: prescription process, self-administration, final consent (or advance consent)
    - What was the process after your family member's death?

Sources, operation, navigation and integration of MAiD regulation

- [Looking back at whole process].
- How did you/your family member know what steps you needed to go through in seeking MAiD? For example, if you/they were not sure about what came next in the process, what did you do?
    - Prompts: guided by physician/nurse practitioner, care coordination service, others?
    - Prompts: What was your role/your family member's role in progressing the process – who was 'driving' the procedural aspects of the process: the health professionals, patient, family? To what extent did you/your family member feel like you/they were in control of the process?
- What did you/they do, or would you/they do, if you/they were unsure of the process or what was permitted or not permitted? Where would you/they look (or did look) or who would you/they (or did you) ask?
    - Prompt: Guided by physician, care groups, contact point at hospital, advocacy group, other?
    - Prompts: If participant discusses information sheets or policies etc. ask whether useful and why.
- Were there any roadblocks? Disagreements? Difficulties accessing MAiD? What was done to get past this?
    - Prompt: Explore especially if one or more physician/nurse practitioners/pharmacists refused to participate.
    - Prompt (if not covered): [If in an institutional setting] - Did your facility facilitate access to MAiD or was it a barrier to access? How? Explicit or implicit? (e.g. Institutional objection).
    - Prompt (if disagreement or barrier): Explore if they initiated complaint process, formal or informal, and how this was initiated.

- What would have helped to make navigating the MAiD system easier?
    - Prompt: Not just individuals, but what is missing in the MAiD system?
- After the process was finished, did you have any contact with anyone about how the MAiD system worked? Did you provide any feedback (positive or negative)? E.g. how it was handled by institution or health professional?
- Have you had any follow up in terms of support with grief and bereavement?
- How has your experience with MAiD and any grief/bereavement shaped or changed your behaviour and attitudes?
    - Prompt: Some participants feel motivated to be involved in advocacy after their experience, whereas others feel quite isolated and as though they can't speak about it. Where do you sit?

Perceptions about MAiD regulation generally

- [Shifting to some more big picture questions now].
- Overall, what was the biggest challenge you/your family member faced in navigating the MAiD system?
    - Prompt: If you could fix one thing with the current system, what would it be?
- Overall, what was the best thing in the MAiD system process?
    - Prompts: What was the key part of the process? If there was one thing that you think is critical for the system running well, what is that?
- We have spoken a lot about the *processes* of MAiD but what about the rules about who can have access to it in the first place (explain eligibility criteria if required). Is this the right group who should have access? (Especially discuss with individuals/families of patients found ineligible).
- MAiD involves a system that has safeguards to ensure only those who are eligible have access to MAiD (safeguarding vulnerable and wider community) while facilitating access for those who qualify (a choice for persons who are suffering from a grievous and irremediable medical condition). So, there is a balance between safe processes that ensure only eligible people access MAiD – but a system that is workable so people can in fact access MAiD. How do you think the current MAiD system strikes this balance?
    - From your experience, is access too easy or too hard, or appropriate?

Demographic information about individual who sought MAiD

For this research to properly understand how the MAiD system is working, we need to make sure we talk to people with diverse experiences and backgrounds. Would it be OK to ask you some questions about yourself and your family member so we can understand your perspectives on what we discuss? Please feel free to say "pass" on any you don't wish to answer.

Questions about participant

- Age
- Gender
- Relationship to family member/patient [if not already mentioned]

Questions about family member/patient

- Age
- Gender
- Marital/relationship status
- Highest educational level
- Occupation
- Country of birth
- Location of residence (e.g. city, town, rural)
- Illness, disease or medical condition
- Other relevant medical conditions
- Cultural background (including ethnicity, religion, if comfortable sharing)
- Primary place of care (and place of administration, if different)
- When MAiD sought
- Outcome of MAiD

Wrapping up

[Discussion about opportunity to review transcript; receiving findings; check-in about well-being and supports/resources for participants]
