# Supplementary material for: A qualitative study of experiences of institutional objection to medical assistance in dying in Canada: ongoing challenges and catalysts for change
Source: BMC Med Ethics. 2023 Sep 21;24:71. doi: 10.1186/s12910-023-00950-9 (PMC10512474; doi:10.1186/s12910-023-00950-9)
Supplement: Supplementary file 2 — Supplementary Material 2 [file 12910_2023_950_MOESM2_ESM.pdf]

OVERVIEW OF INTERVIEW GUIDE

- INDIVIDUALS -

Introduction

- Introduce interviewer(s) and study.
- Thank you for being able to help with this research. Before we go into detail, can we just first deal with the administrative side of things?
- [Zoom recording, consent, and confidentiality discussion].
- You may feel that some of the questions I ask are stressful or upsetting. If you do not want to answer any of these questions, please just say so. There are no right and wrong answers; we are simply interested in your views.
- Do you have any questions for me before we start the interview?

Overview of individual seeking MAiD

Before we go on to talk about your experiences of making decisions about MAiD and the process, can we just briefly talk about your illness and where you are now? Are you okay with that? [If appropriate, a chance to understand a bit about the person's approach to managing their condition and decision-making].

Remaining interview approach

In terms of the rest of our discussion, I know we have a lot to cover, and I want to make sure we can capture it all. To start let's step through the MAiD process in order so you can comment on each part of the process. I will then finish with some more general and open-ended questions which will also provide a chance for you to tell me other things. Is it OK if we proceed that way and perhaps if am a bit directive to make sure we understand your experience?

First discussion of MAiD

- What was your exposure to MAiD (if any) prior to your own experience?
- When did you first become aware of MAiD and that it might be possible? How did this topic come up (e.g. sources of information)?
  - Prompts: Who did you talk to about this?
  - Why did you go down this path of seeking information about MAiD? (Motivation, what you wanted).
  - [If involved in prior MAiD advocacy or reform, ask further information about nature of involvement and motivations for involvement in this work].
- Was it easy to get information you wanted about MAiD or were there barriers?
  - Prompt: Where did you go for information?

- When did you first talk about MAiD with a physician or nurse practitioner (or other health professional/care coordinator)? Who raised it? How did the health professional respond?
    - Prompt: Were there any barriers or concerns with your physician, nurse practitioner, or other health professionals?

Assessment process

- Can you tell us when you sought MAiD [and which track you were on (if post March 2021)]?
- What was your experience of requesting and being assessed for MAiD? How did the process start?
    - Prompts: Were you able to easily find a physician/nurse practitioner to guide you through the process? Were you connected to a care coordinator or other service?
    - Can you recall whether you had to make a written request to a physician or nurse practitioner to start the process? How did this work?
    - Were there any difficulties in getting a witness (or two if pre-March 2021) for this written request?
    - Who located the health professionals to do the assessments? Were there any difficulties in finding two independent physicians/nurse practitioners to confirm you were eligible?
    - Were there any difficulties with eligibility criteria? [Particularly ask about specific eligibility criteria if mentioned as an issue].
        - [Note after March 2021, reasonably foreseeable natural death is no longer an eligibility criterion but does determine whether a person is Track 1 or Track 2].
    - Do you recall how long the process took from the written request to when you were found eligible for MAiD?
- General prompts:
    - Did you need to contact any care coordination services?
    - Did you ever use telehealth for MAiD consultations?
    - Were there issues with travel to access health professionals for assessments or for the provision of MAiD?
    - Where were you living when seeking MAiD? Did your facility facilitate access to MAiD or was it a barrier to access? How? Was this explicit or implicit? [e.g. institutional objection].
    - What do you think about the waiting period? [Note prior to March 2021, 10-day waiting period and after March 2021, 90-day period for Track 2 (when natural death not reasonably foreseeable), but potential to be shortened if loss of capacity imminent].

    - [If Track 2 (post March 2021, natural death is not reasonably foreseeable) and neither assessor/providers have expertise in the illness/condition]: Were there any difficulties finding an independent practitioner with expertise in the condition causing your suffering to confirm eligibility? [Note this would often be arranged by clinicians/care coordinators].
- Overall, looking back at the request and assessment process, was the process straightforward? Challenging? Why?
    - Prompts: What parts of the process worked well? What parts (if any) needed improvement?

Prescription of medication and provision of MAiD for eligible MAiD patients

<u>If plan to have provider administered MAiD:</u>

- Will you choose to have provider-administered MAiD? Was there any discussion of self-administration as an alternative? If so, what were you told and what impression were you left with about it?
- The choice about whether or not to have MAiD it and when to have MAiD – that is a choice. What is guiding you about your decision to have MAiD and the timing of that?
- [If after March 2021 and Track 1 (natural death reasonably foreseeable)]: Have you considered a final consent waiver? What was your experience of this (in terms of making the written arrangement)?

<u>If plan to have self-administered MAiD:</u>

- When will you obtain the medication?
    - How do you think you will feel when you obtain it?
- Have you considered advance consent for failed self-administration? What was your experience of this (in terms of making a written arrangement)?

Sources, operation, navigation and integration of MAiD regulation

- [Looking back at whole process].
- How did you know what steps you needed to go through in seeking MAiD? For example, if you were not sure about what came next in the process, what did you do?
    - Prompts: guided by physician/nurse practitioner, care coordination service, others?
- What did you do, or would you do, if you were/are unsure of the process or what was permitted or not permitted? Where would you look (or did look) or who would you (or did you) ask?
    - Prompt: Guided by physician, nurse practitioner, care groups, contact point at hospital, advocacy group, other?

- Prompts: If participant discusses information sheets or policies etc. ask whether useful and why?
- Were there any roadblocks? Disagreements? Difficulties accessing MAiD? What was done to get past this?
  - Prompt: Explore especially if one or more physician/nurse practitioners/pharmacists refused to participate.
  - Prompt (if not covered): [If in an institutional setting] - Did your facility facilitate access to MAiD or was it a barrier to access? How? Was this explicit or implicit? (e.g. Institutional objection).
  - Prompt (if disagreement or barrier): Explore if they initiated complaint process, formal or informal, and how this was initiated.
- What would have helped to make navigating the MAiD system easier for you?
  - Prompt: Not just individuals, but what is missing in the MAiD system?
- Have you had any contact with anyone about how the MAiD system has worked so far? Did you provide any feedback (positive or negative)? E.g. how it was handled by institution or health professional?

Perceptions about MAiD regulation generally

- [Shifting to some more big picture questions now].
- Overall, what was the biggest challenge you faced in navigating the MAiD process?
  - Prompt: If you could fix one thing with the current system, what would it be?
- Overall, what was the best thing in the MAiD process?
  - Prompts: What was the key part of the process? If there was one thing that you think is critical for the system running well, what is that?
- We have spoken a lot about the *processes* of MAiD but what about the rules about who can have access to it in the first place (explain eligibility criteria). Is this the right group who should have access? (Especially discuss with individuals found ineligible).
- MAiD involves a system that has safeguards to ensure only those who are eligible have access to MAiD (safeguarding vulnerable and wider community) while facilitating access for those who qualify (a choice for people who are suffering from a grievous and irremediable medical condition). So, there is a balance between safe processes that ensure only eligible people access MAiD – but a system that is workable so people can in fact access MAiD? How do you think the current MAiD system strikes this balance?
  - From your experience, is access too easy or too hard, or appropriate?

Demographic information about individual who sought MAiD

For this research to properly understand how the MAiD system is working, we need to make sure we talk to people with diverse experiences and backgrounds. Would it be OK to ask you some questions about yourself so we can understand your perspectives on what we discuss? Please feel free to say “pass” on any you don’t wish to answer.

- Age
- Gender
- Marital/relationship status
- Highest educational level
- Occupation
- Country of birth
- Location of residence (e.g. city, town, rural)
- Illness, disease or medical condition
- Other relevant medical conditions
- Cultural background (including ethnicity, religion, if comfortable sharing)
- Primary place of care (and place of administration, if different)
- When MAiD sought
- Outcome of MAiD

Wrapping up

[Discussion about opportunity to review transcript; receiving findings; check-in about well-being and supports/resources for participants]
