# Supplementary material for: A qualitative study of experiences of institutional objection to medical assistance in dying in Canada: ongoing challenges and catalysts for change
Source: BMC Med Ethics. 2023 Sep 21;24:71. doi: 10.1186/s12910-023-00950-9 (PMC10512474; doi:10.1186/s12910-023-00950-9)
Supplement: Supplementary file 3 — Supplementary Material 3 [file 12910_2023_950_MOESM3_ESM.pdf]

OVERVIEW OF INTERVIEW GUIDE

- ORGANIZATIONAL DECISION-MAKERS (MAiD TEAM MEMBERS) -

The interview guide will also be nuanced for different experiences of health professionals.

Introduction

- Introduce interviewer(s) and study.
- Thank you for being able to help with this research. Before we go into detail, can we just first deal with the administrative side of things?
- [Zoom recording, consent, and confidentiality discussion].
- As you would have gathered from the consent form, the purpose of today's interview is to hear about your views and experiences with MAiD. You may feel that some of the questions I ask are stressful or upsetting. If you don't want to answer any of these questions, please just say so. There are no right and wrong answers; we are simply interested in people's views.
- Likewise – we are trying to capture rich descriptions and perspectives so please use case examples where this is illustrative but de-identify these as much or as little as you need to, to protect privacy.
- Do you have any questions for me before we start the interview?

1. Role in regulating ('shaping or steering') MAiD practice

- What is your position and involvement with MAiD practice or regulation (now or in the past)
- What role does your organization play in MAiD practice?
    - Prompts: may be direct regulation of MAiD or indirect through regulating health professionals generally
    - [discussion of meaning of regulate – influence, steer behavior]

2. Organisational context

- Can you provide a general overview of how MAiD operates within the institution/health authority/organization
    - Scope of MAiD services available
    - Composition of institution/client base
    - Location
    - Interactions with provincial oversight/other bodies that regulate or monitor

- Reflect on the change of practice when MAiD was introduced

    - What issues were important?
    - What has worked well?
    - What were the challenges?

- Now – 5-6 years on
    - Reflect on changes – what has improved, what is working well, what are the ongoing challenges?

- What steps, if any did your organization take to respond to the Bill C-7 amendments?

3. Scope of regulation

- Who does your organization seek to regulate or influence?
    - Any specific groups concerned with protecting? (eg vulnerable patients, practitioner wellbeing)
- If in hospital/institution/health authority
    - What is the organization's role in deciding who is eligible to assess/provide MAiD?
    - Interaction with other staff/health professionals
    - Patients/families
    - Other bodies
- What "tools of regulation" (i.e. means of shaping or steering practice) does your organization have at its disposal?
    - Prompts: law, policy, guidelines, ethical codes, training, professional norms, anything else?
    - Any insight into the development of tools, aids, educational programs?
- How are those tools of regulation used?
    - Prompts: all at same time? Elevation of tools as needed? More indirect influence or directing behavior through sanctions?
- What people or groups give effect to or implement those tools of regulation? How is this done?
    - Prompts: on the ground engagement?
    - Arms-length issuing of policies?
    - Proactive engagement (promoting training) or
    - Reactive (disciplinary role if required)?

4. Regulation in practice

- What aspects of your organization's regulation are working well? Why?
- What aspects of your organization's regulation are not working well? Why?
- Probe (if not covered): internal processes and systems
- Which people's or groups' behavior is challenging to guide?

- Do you have sufficient power and influence to fulfil your organization's regulatory role?
- How do you manage any competing or conflicting guidance from other regulators?

- *Hospital/institution/health authority*
    - Any instances of receiving/implementing feedback?
    - Revision of policies? (impetus behind revision)
    - Delivery of education/training to staff
    - Support programs
    - Examples of issues with compliance or ignorance of obligations within the hospital
    - Any examples of difficulties in managing different people's behaviour
    - Any issues with individual conscientious objection among staff/volunteers (including details around management, if applicable)
    - Reflection on the creation of a MAiD-specific role (e.g. MAiD coordinator role was put into place)
    - Have you been involved with addressing institutional objections and/or transfers?

5. If appropriate, talk through aspects of MAiD process

6. Perceptions about MAiD regulation generally

- What works well with the current regulation of MAiD overall (stepping past your organization's role)?
- How could current regulation of MAiD be improved?
    - What matters are currently regulated that shouldn't be?
    - What matters aren't currently regulated that should be?
- MAiD involves a system that has safeguards to ensure only those who are eligible have access to MAiD while facilitating reasonable access for those who qualify. How do you think the current MAiD regulation strikes this balance?
- If you were designing the best possible system of MAiD regulation, what principles or values do you think should underpin that system?
    - What features do you think the system should have?
- What is the most effective way to guide health professionals' actions in this area?

Demographic information

For this research to properly understand how the MAiD system is working we need to make sure we talk to people with diverse experiences and backgrounds. Just to wrap up I'd like to ask you some demographic questions. Please feel free to say "pass" on any you don't wish to answer.

i. What is your age? ____

ii. Gender identity:
- ☐ Male
- ☐ Female
- ☐ Other

iii. Employer/organization affiliated with:

____

iv. Employment status:
- ☐ Full time
- ☐ Part/time or casual
- ☐ Studying
- ☐ Not employed
- ☐ Other ____________________________________

i. Location: ____
(e.g. city, town, rural)

v. Anything about your cultural background (including ethnicity, religion) you wish to share? ____

____

____

Wrapping up

We are giving people the option to **review their transcript**, if they would like to do so. We will send the transcript to you once it is completed and you can choose to check it for accuracy or to add any comments if you would like, but you don't have to.

Your views on this issue are really important to us and we want to make sure they are reflected fully in our findings. If you think of anything else that you would like to add, please feel free to get in contact with us to let us know.

Expert roundtables

If any aspect of this interview has caused you distress, the Participant Information Sheet includes some support options you are available to contact.

Do you have any questions of me?

Thank you very much for taking part in this interview.
