# Supplementary material for: A qualitative study of experiences of institutional objection to medical assistance in dying in Canada: ongoing challenges and catalysts for change
Source: BMC Med Ethics. 2023 Sep 21;24:71. doi: 10.1186/s12910-023-00950-9 (PMC10512474; doi:10.1186/s12910-023-00950-9)
Supplement: Supplementary file 4 — Supplementary Material 4 [file 12910_2023_950_MOESM4_ESM.pdf]

DALHOUSIE UNIVERSITY

QUT

OVERVIEW OF INTERVIEW GUIDE

- MAiD Assessors and Providers –

Introduction

- Introduce interviewer(s) and study.
- Thank you for being able to help with this research. Before we go into detail, can we just first deal with the administrative side of things?
- [Zoom recording, consent, and confidentiality discussion].
- As you would have gathered from the consent form, the purpose of today's interview is to hear about your views and experiences with MAiD. As an experienced health professional, I know that death and dying are part of your job, but nevertheless, you may feel that some of the questions I ask are stressful or upsetting. If you don't want to answer any of these questions, please just say so. There are no right and wrong answers; we are simply interested in people's views.
- Likewise – we are trying to capture rich descriptions and perspectives so please use case examples where this is illustrative but de-identify these as much or as little as you need to, to protect privacy.
- Do you have any questions for me before we start the interview?

1. Overall experience with end-of-life decision-making and medical assistance in dying (MAiD)

- What is your role [if not known]
- Could you please describe how you decided to participate in MAiD?
- Describe briefly your experience/background with MAiD and how it became/is part of your clinical practice?

2. Source of information about steps in the process

- How did you find out about the steps in the MAiD assessment process? What sources of information?
- How did you find out about reporting requirements?
- What sources guide you in making decisions about MAiD?
    - Prompts: law, policy, guidelines, ethical codes, training, professional norms, anything else?
    - How do they guide you?
    - Is their guidance useful for you when making decisions about MAiD?
- What people or institutions guide you in making decisions about MAiD?

  - Prompts: colleagues, ethics committees, MAiD community of practice, College, [refer to relevant provincial services - E.g. MAID Care Co-ordination Service in Ontario], CMPA (the "insurer") (or CNPS for nurse practitioners)
  - How do they guide you?
  - Is their guidance useful for you when making decisions about MAiD?
- How influential are those sources, people and institutions in how you make decisions about MAiD?
  - Prompt: Which is most important?
- How useful are the above sources, people or institutions when making decisions about MAiD? How do you manage any competing or conflicting guidance in these sources, people or institutions?
  - Prompts: Describe a circumstance where a policy or professional standard shaped your decision. Conversely, describe a circumstance (if any) where policy was not determinative.
- Where there is disagreement or uncertainty about the processes to be followed or whether someone is eligible to access MAiD, what steps would you take to resolve that disagreement or uncertainty?

3. Initiating MAiD

- How does a MAiD case begin for you?
  - Have patients seeking MAiD raised this topic with you? How?
- How did you decide when you had a request for MAiD?
- Once you had a first request for MAiD, how did you know what steps to take in the assessment process?
- Are there any aspects of MAiD that you have a conscientious objection to or do not feel comfortable participating in – eg will assess but not provide, etc
- If you have a conscientious objection to MAiD, how did you raise this? Did you feel supported in declining to participate in MAiD?

4. Assessment process and eligibility requirements

- What has been your experience of the assessment process in terms of what is working and what is not working?
  - Prompts: independent assessments, consultation with relevant practitioners (expertise), second assessment, safeguards (e.g. two tracks, timeframes), MAiD reporting
- Now I'd like to focus on the eligibility requirements for accessing MAiD, could you tell me about your experience applying these requirements (what's working/what is not working; why?)
- Prompts if not covered above:
  - How have you found applying residence requirements?
  - Eligibility for healthcare in Canada?

- Have you had any difficulties making judgments about whether a person has a grievous and irremediable medical condition?
  - Serious illness, disease, or disability (excluding mental illness until 17 March 2023)
  - Advanced state of irreversible decline
  - Experience unbearable physical or mental suffering from an illness, disease, disability or state of decline that cannot be relieved under conditions that the person considers acceptable
  - (Prior to March 2021, about whether a death was reasonably foreseeable?).
- How confident do you feel to make judgments about whether a person has a grievous and irremediable medical condition? How do you overcome any potential uncertainty?
- How confident do you feel in making judgments about whether the request is voluntary and free of external pressure? How do you overcome any potential uncertainty?
- Have you experienced any issues in relation to assessing capacity?
- How do you approach informed consent?
- How have you dealt with the "reasonably foreseeable natural death" (RFND) requirement under the two tracks?
  - How have the legally required wait times for those with a RFND operated in practice (ie previously the requirement was 10 days, now there is a 90-day requirement in relation to deaths that are not reasonably foreseeable)?
  - Dealt with patients without a RFND?
- Is it difficult / easy to find another independent clinician [ie doctor/nurse practitioner] to do the other assessment? Why?
- Was conscientious objection (or other reasons for not participating) a problem at any stage of the MAiD process?
- Have you experienced any issues with institutions which object to MAiD at any stage of the MAiD process?
- Other issues to prompt:
  - regional/remote access;
  - telehealth;

5. Reporting requirements

- Could you please talk about your experience navigating the reporting requirements for MAiD in practice (works, not works? Why?)
- Prompts if not covered above
  - What do you believe are the main benefits/issues/challenges going through the MAiD reporting processes?

    - Do you believe these processes are justified – why? Or why not?
    - Any examples of data that is not captured or reported on that should be?

6. Prescription of medication and provision of MAiD for eligible MAiD patients

- How did you navigate the process for prescribing or administering the MAiD medication?
- How was the method of MAiD chosen (ie self-administration or provider administration)?
- Key prompt: How is the difference between self-administration and provider administration working in practice? How is this decision made?
- Any issues in locating witnesses? (at any stage of the process)
- What aspects of the process for providing MAiD work well and what needs improvement?
    - Prompts: prescription process, self-administration vs provider administration, final consent waiver, advance consent for failed self-administration
- What about after the patient has died – staying with how the system works rather than clinical issues – should the law or regulatory framework do anything different?
- Any issues with conscientious objection from other healthcare professionals (e.g., pharmacist is key for dispensing) or institutional objections? (At any stage of the process?)

7. The regulatory system's ability to meet policy goals in practice

- What is the best aspect of the MAiD process/structure and what needs the most improvement?
- How could current regulation of MAiD be improved? What are barriers?
    - What matters are currently regulated that shouldn't be?
    - What matters aren't currently regulated that should be?
- We have spoken a lot about the *processes* of MAiD but what about the rules about who can have access to it in the first place (explain eligibility criteria). Is this the right group who should have access under the law?
- The key policy goals underpinning the MAiD law appear to be:
    - Respecting personal autonomy – providing choice for grievously and irremediably ill patients.
    - Alleviating suffering
    - But also: Safeguarding individuals who need protection and the wider community.
- MAiD involves a system that has safeguards to ensure only those who are eligible have access to MAiD while facilitating reasonable access for those who qualify. How do you think the current MAiD regulation strikes this balance?
- Changes made with Bill C-7 – any observations about how this is working since the changes came into force in March 2021?

8. Health professional steering or shaping the MAiD system

- Have you tried to make changes or improvements to the MAiD system
- In what way?
- What changes are the most important?

9. Best way to guide behaviour

- Next is a question that is a little abstract or theoretical. The government wants health professionals to follow rules for providing MAiD so the federal and provincial governments have set up various rules: i.e. MAiD laws, policies and guidelines, and there are guidelines issued by organizational regulators (including Colleges). If you are trying to design a system that health professionals like you will listen to and follow, what is the most effective way to guide behavior when providing MAiD? What sorts of 'rules' are health professionals most likely to listen to, what will they follow in providing MAiD? [Prompt: law, policy, guidelines, ethical codes, training, professional norms, anything else? Which of these are most likely to influence your decisions about providing MAiD?]

10. Macro observations

- Putting aside the current MAiD system you have been involved in, if you could tell those designing a new MAiD system who wanted it to be the best system possible some advice, what advice what you have?
    - What principles or values do you think should underpin that system?
    - What features do you think the system should have?
    - This is about guiding people's behavior - what is the most effective way to guide people in this area?

Demographic information

For this research to properly understand how the MAiD system is working we need to make sure we talk to people with diverse experiences and backgrounds. Just to wrap up I'd like to ask you some demographic questions. Please feel free to say "pass" on any you don't wish to answer.

i. How many MAiD cases involved in either as assessor or provider? ______________

ii. Age: ____

_

iii. Gender:
- ☐ Male
- ☐ Female
- ☐ Other

iv. Location of primary practice: ____
(e.g. city, town, rural)

ii. Setting of primary practice:
(select all that apply)
- ☐ Hospital
- ☐ Hospice
- ☐ Aged Care/Long Term Care
- ☐ At home
- ☐ Other ______________________________________
- ☐ Public
- ☐ Private
- ☐ Religious/type_______________________________

i. Employment status:
- ☐ Full time
- ☐ Part/time or casual
- ☐ Studying
- ☐ Not employed
- ☐ Other ____________________________________

ii. Health profession:
- ☐ Specialist
- ☐ Physician
- ☐ Resident
- ☐ Nurse practitioner
- ☐ Pharmacist
- ☐ Allied health professional
- ☐ Other (please specify)

____

iii. Main are your main area(s) of practice:

- ☐ Anesthesiology
- ☐ Cardiology
- ☐ Dietetics
- ☐ Emergency Medicine
- ☐ Exercise physiology
- ☐ Family medicine
- ☐ Gastroenterology
- ☐ Internal Medicine
- ☐ General practice
- ☐ Geriatrics
- ☐ Hematology
- ☐ Intensive Care
- ☐ Medical Oncology
- ☐ Radiation Oncology
- ☐ Neurology
- ☐ Occupational therapy
- ☐ Palliative Care
- ☐ Paramedicine
- ☐ Pharmacy
- ☐ Physiotherapy
- ☐ Prosthetics/orthotists
- ☐ Psychiatry
- ☐ Psychology
- ☐ Radiology
- ☐ Radiography
- ☐ Respiratory
- ☐ Renal
- ☐ Social work
- ☐ Speech pathology
- ☐ Surgery
- ☐ Prefer not to say
- ☐ Other (please specify) ____
- ☐ Additional areas of practice (please specify) ____

iv. Years experience/practice: ____

iii. Anything about your cultural background (including ethnicity, religion) you wish to share? ____

____
